# Chronic ingestion of soy peptide supplementation reduces aggressive behavior and abnormal fear memory caused by juvenile social isolation

**DOI:** 10.1038/s41598-024-62534-w

**Published:** 2024-05-21

**Authors:** Hideki Tamura, Akiko Miyazaki, Takashi Kawamura, Hikaru Gotoh, Naoki Yamamoto, Minoru Narita

**Affiliations:** 1 Laboratory of Biofunctional Science, School of Pharmacy and Pharmaceutical Sciences, Hoshi University, 2-4-41 Ebara, Shinagawa, Tokyo, 142-8501 Japan; 2 Institute for Advanced Life Sciences, Hoshi University School of Pharmacy and Pharmaceutical Sciences, Tokyo, Japan; 3 Department of Pharmacology, Hoshi University School of Pharmacy and Pharmaceutical Sciences, Tokyo, Japan; 4 Department of Biology, Faculty of Science, Kyushu University, Fukuoka, 819-0395, Japan; 5 Department of Pharmacy, National Cancer Center Hospital, Tokyo, Japan

**Keywords:** Stress and resilience, Social behaviour, Fear conditioning

## Abstract

Juvenile loneliness is a risk factor for psychopathology in later life. Deprivation of early social experience due to peer rejection has a detrimental impact on emotional and cognitive brain function in adulthood. Accumulating evidence indicates that soy peptides have many positive effects on higher brain function in rodents and humans. However, the effects of soy peptide use on juvenile social isolation are unknown. Here, we demonstrated that soy peptides reduced the deterioration of behavioral and cellular functions resulting from juvenile socially-isolated rearing. We found that prolonged social isolation post-weaning in male C57BL/6J mice resulted in higher aggression and impulsivity and fear memory deficits at 7 weeks of age, and that these behavioral abnormalities, except impulsivity, were mitigated by ingestion of soy peptides. Furthermore, we found that daily intake of soy peptides caused upregulation of postsynaptic density 95 in the medial prefrontal cortex and phosphorylation of the cyclic adenosine monophosphate response element binding protein in the hippocampus of socially isolated mice, increased phosphorylation of the adenosine monophosphate-activated protein kinase in the hippocampus, and altered the microbiota composition. These results suggest that soy peptides have protective effects against juvenile social isolation-induced behavioral deficits via synaptic maturation and cellular functionalization.

## Introduction

Chronic social isolation (SI) is a highly stressful situation for gregarious animals and humans^1^ and causes severe emotional and psychological harm and cognitive deterioration^2^. In particular, during juvenility and adolescence, the deprivation of social experience due to peer rejection is detrimental to adult brain function^3^ and is implicated in the etiology of neuropsychiatric disorders, including autism spectrum disorder, attention deficit/hyperactivity disorder (ADHD), schizophrenia, and depression^4–6^. Because early to late adolescence is the period of critical reorganization of brain neural networks, including in the medial prefrontal cortex (mPFC), which are sensitive to environmental factors, such as stress and malnutrition^7,8^, early intervention during this period may reduce the onset and development of these disorders in adulthood.

A growing body of evidence indicates that juvenile SI after weaning induces various neurochemical, physiological, and behavioral abnormalities in rodents^6,9^. Mice reared in isolation exhibit increased aggression and impulsivity, hyperactivity in novel environments, aberrant social behavior, prepulse inhibition deficit, depression-like behavior, and cognitive impairment^10–15^. These abnormal behaviors are associated with alterations in the molecular, neuroanatomical, and neuronal excitability of the brain regions corresponding to emotions. For example, SI affects excitatory and inhibitory synaptic transmission; the monoaminergic system; and neurotrophin and neuropeptide expression in the mPFC, amygdala, hypothalamus, and hippocampus^16–19^. In addition, it leads to a decrease in pyramidal neuron spine density and impaired dendritic spine maturation and myelination in mPFC^15,20,21^ as well as to a reduction in neurogenesis in the hippocampus^13^. Furthermore, post-weaning SI causes a reduction of synaptic excitability in the mPFC in adulthood^22^ and hyperactivation of the hippocampus^23^. Interestingly, preventing these changes could ameliorate post-weaning SI-induced sociability deficit and aggression^24,25^. These data indicate that synaptic abnormality in mPFC and hippocampal dysfunction due to deprivation of social contact during juvenility could be responsible for some emotional behavioral changes in adulthood.

Nutrition, including nutritional supplements, is recognized to play an important role in the development of brain functions and structures such as cognitive and emotional networks, especially during adolescence^26^. Some nutritional supplements, such as fish oils, fatty acids, micronutrients, and soy peptides, have been reported to improve emotional and cognitive brain function^27–30^. Peptides from soybean hydrolysate are particularly interesting in this regard because they are absorbed from the small intestine into the blood without degradation, reach the brain through the blood–brain barrier, and highly concentrated in the forebrain, hippocampus, striatum, and hypothalamus^31–33^, indicating the possibility of direct modulation of neuronal signaling pathways by soy peptides. Consistent with this finding, the activation of synaptic plasticity signaling by soy peptides has been shown in aging-related cognitive dysfunction in mice^34^. It has been suggested that soybean-derived glycine-arginine dipeptide promotes neurogenesis in the hippocampus and cerebral cortex^32^; therefore, soy peptides could reorganize neural networks, thereby, improve recognition memory and cognitive dysfunction^27,28^. Moreover, soy peptides affect central monoamine transmission systems, in addition to exerting anxiolytic-like effects in adult mice^35,36^.

Based on these neuromodulatory effects of soy peptides, we hypothesized that soy peptides could mitigate detrimental alterations in neuronal function induced by post-weaning SI, resulting in the reduction of emotional abnormalities. Therefore, in this study, we aimed to examine the effects of soy peptides on aggression, impulsivity, and fear memory in isolation-reared mice using a dyadic social interaction test, variable time wait task, and contextual fear conditioning and investigated whether soy peptides influenced synaptic-associated proteins synaptophysin and postsynaptic density 95 (PSD95) in the mPFC, and adenosine monophosphate-activated protein kinase (AMPK), a soy peptides response signaling molecule^37^ and the downstream synaptic plasticity marker cyclic adenosine monophosphate response element binding protein (CREB) in the hippocampus. Given previous evidence for a potent influence on neuronal function through the interaction between the gut microbiota and the brain^38^, we further aimed to determine whether the composition of the microbiota was altered by the ingestion of soy peptides.

## Results

### Soy peptide ingestion ameliorates aggression, but not impulsivity, in SI mice

**Figure 1:**
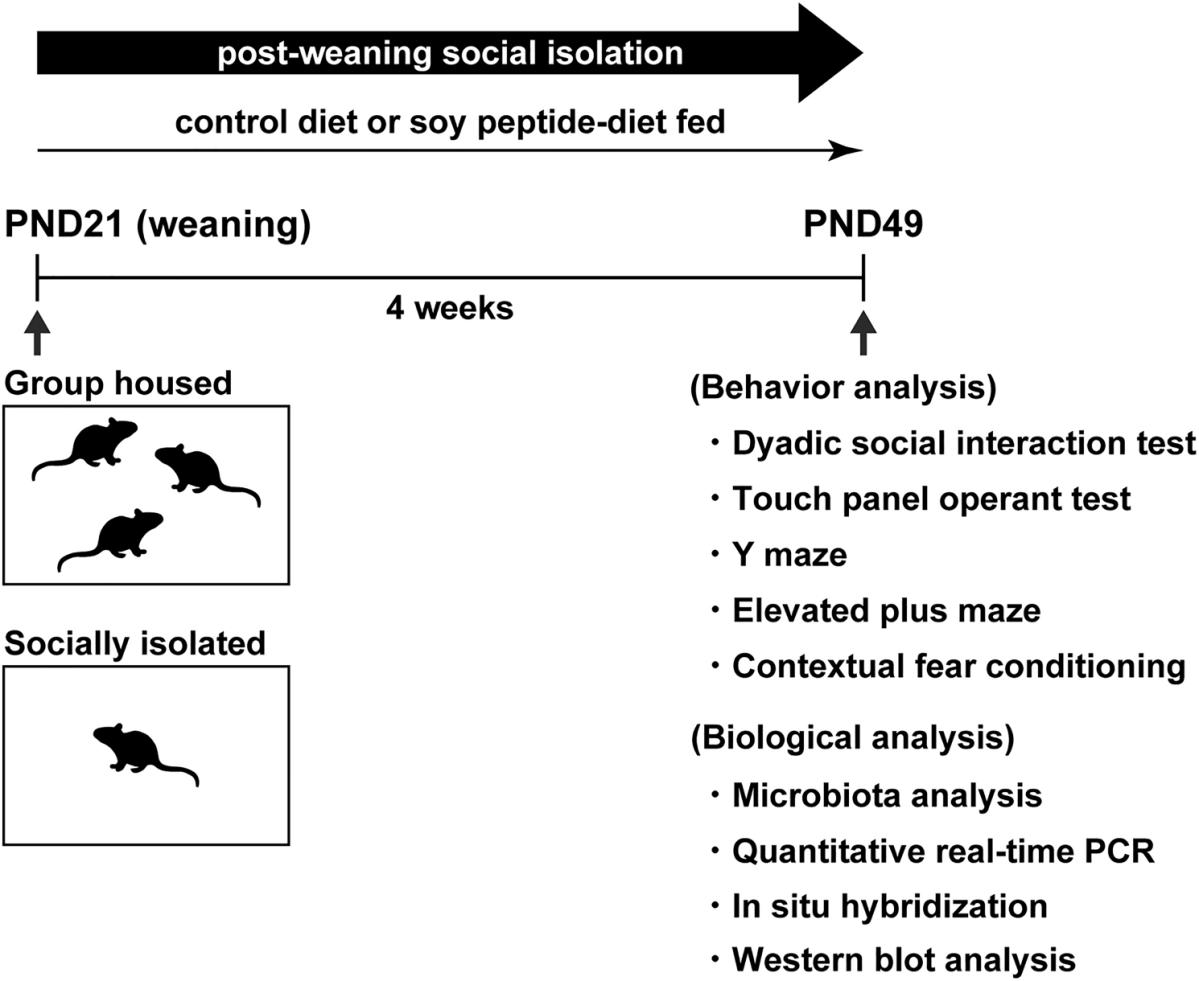
Experimental schedule. Mice were group-housed (3 mice/cage) or socially isolated from weaning and fed either a control diet or a soy peptide diet until the end of the experiments. After 4 weeks of social isolation and feeding a soy peptide diet, a dyadic social interaction test, touch panel operant test, Y maze, elevated plus maze, and contextual fear conditioning were performed. Other mice were used for microbiota analysis, quantitative real-time PCR, in situ hybridization, and western blot analysis. Different mice were used in each experiment.

Group-house (GH) and SI mice were fed a diet consisting of soy peptides or a control diet for 4 weeks post-weaning (Fig. 1), thereby constituting the four study groups (control diet-fed GH, soy peptide-diet fed GH, control diet-fed SI, and soy peptide-diet fed SI mice). The body weight gain and daily food consumption did not differ significantly among these four groups, i.e., no significant main effects of diet (body weight gain: *F*_(1, 20)_ = 0.150, *p* = 0.6989; food consumption: *F*_(1, 20)_ = 1.324, *p* = 0.2635) and rearing (body weight: *n* = 6, *F*_(1, 20)_ = 0.3093, *p* = 0.5843; food consumption: *F*_(1, 20)_ = 0.2409, *p* = 0.6289; Supplementary Table S1).

**Figure 2:**
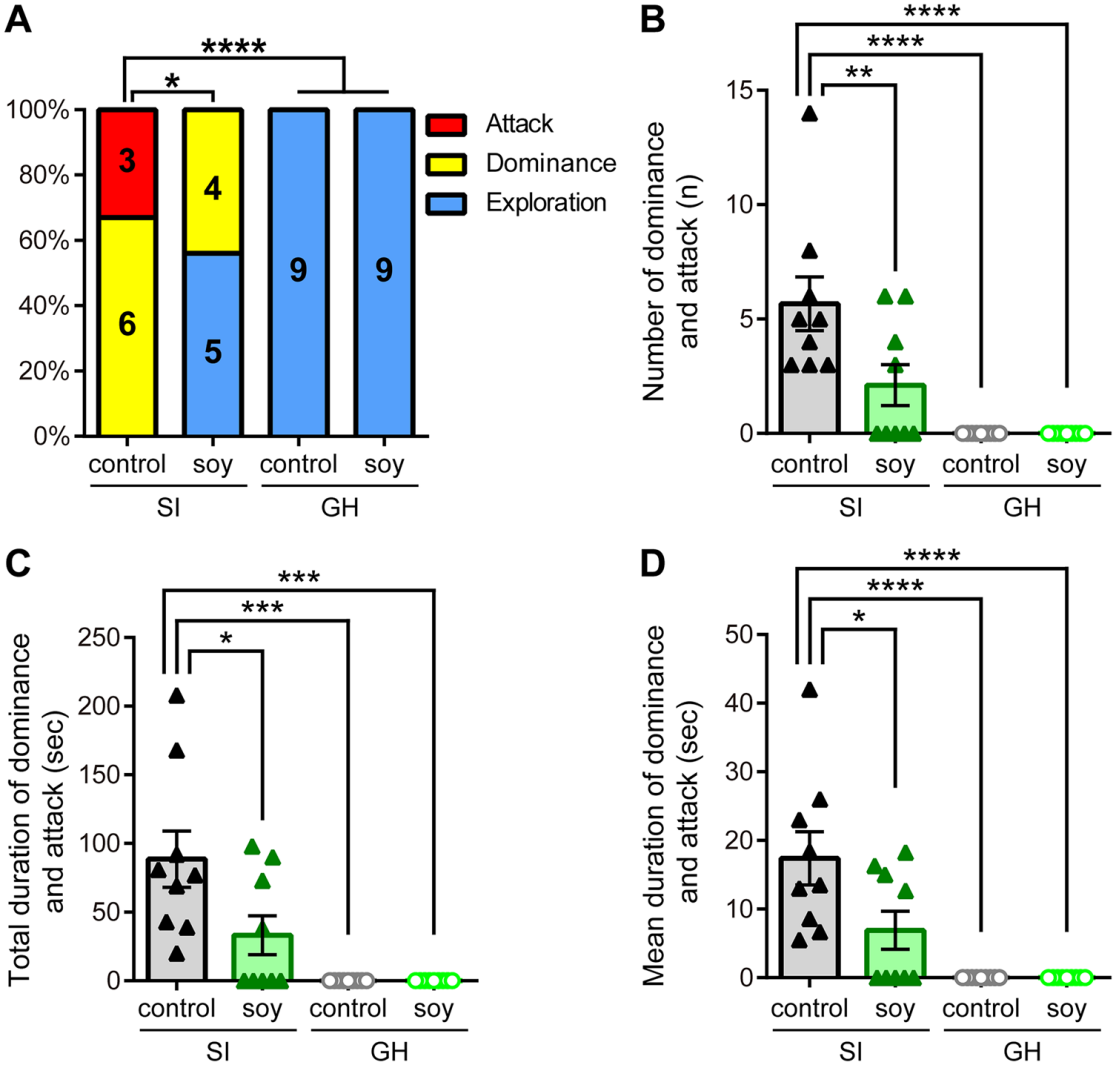
Effects of soy peptides on aggression. **(A)** The numbers and proportions of socially isolated (SI) and group-housed (GH) mice fed with control or soy peptide diet exhibiting only social exploration (blue), social dominance (yellow), or at least one biting attack (red) in the dyadic social interaction test. Digits inside columns indicate the number of samples. *p < 0.05, ****p < 0.0001; Fisher tests. **(B–D)** The number (**B**), total duration (**C**), and mean duration (**D**) of dominance and attack behavior are shown for SI mice given control diet (black closed triangles; *n* = 9) or soy peptide diet (dark green closed triangles; *n* = 9) and GH mice given control diet (gray open circles; *n* = 9) or soy peptide diet (light green open circles; *n* = 9). Among SI mice, soy peptide-fed mice show a significant decrease in offensive aggression toward an unfamiliar mouse when compared with control diet-fed mice. Data are expressed as mean and SEM. *p < 0.05, **p < 0.01, ***p < 0.001, and ****p < 0.0001; two-way ANOVA with Tukey *post*-*hoc* test.

The effect of ingestion of soy peptides on aggression in GH and SI mice was examined in the dyadic social interaction test. The behavioral phenotypes of mice were categorized into one of three categories (i.e., *exploration*, *dominanc*e, and *attack*), according to the degree of aggressive behaviors. All GH mice showed only social exploration toward an unfamiliar male, regardless of the type of diet (18/18; Fig. 2A). However, in the SI group, all control diet-fed mice showed a highly elevated level of aggression (*p* < 0.0001 *vs*. GH control and soy; Fig. 2A): six of nine mice had high social dominance status (6/9; 67%), and the others exhibited attacking behavior towards an unfamiliar mouse (3/9; 33%; Fig. 2A). Control diet-fed mice had more dominance events and longer dominance durations than GH mice (Supplementary Fig. S1A), and attacked an unfamiliar mouse for longer duration (Supplementary Fig. S1B). In contrast, soy peptide-fed SI mice displayed significantly less aggressive behavior (five mice engaged in social exploration [5/9; 56%] and the others engaged in social dominance [4/9; 44%]; *p* = 0.0294 *vs*. SI control; *p* = 0.0824 *vs*. GH soy; Fig. 2A). Soy peptide-fed SI mice had fewer dominance events than mice fed a control diet (*p* = 0.0392 *vs*. SI control; Supplementary Fig. S1A).

Two-way ANOVA for effects on aggressive behavior, including the number, total duration, and mean duration of dominance and attack behavior, showed significant main effects of rearing (number; *F*_(1, 32)_ = 27.76, *p* < 0.0001; total duration: *F*_(1, 32)_ = 23.76, *p* < 0.0001; mean duration; *F*_(1, 32)_ = 25.74, *p* < 0.0001) and diet (number; *F*_(1, 32)_ = 5.802, *p* = 0.0219; total duration: *F*_(1, 32)_ = 4.906, *p* = 0.0340; mean duration; *F*_(1, 32)_ = 4.741, *p* = 0.0369), and a significant interaction between rearing and diet (number; *F*_(1, 32)_ = 5.802, *p* = 0.0219; total duration: *F*_(1, 32)_ = 4.906, *p* = 0.0340; mean duration; *F*_(1, 32)_ = 4.741, *p* = 0.0369; Fig. 2B–D). While the number of aggression and the total and mean duration of aggressive behavior of control diet-fed SI mice were significantly greater than in the GH groups (SI control: number, 5.7 ± 1.1 times, *n* = 9, *q*_32_ = 7.678, *p* < 0.0001; total duration, 88.6 ± 19.4 s, *q*_32_ = 7.089, *p* = 0.0001; mean duration, 17.3 ± 3.7 s, *q*_32_ = 7.251, *p* < 0.0001 *vs*. GH control and soy; Fig. 2B–D), soy peptide-fed SI mice exhibited a significant increase in the number of aggression (SI soy: 2.1 ± 0.8 times, *n* = 9, *q*_32_ = 4.817, *p* = 0.0092 *vs*. SI control; Fig. 2B) and a shorter total and mean duration of threat behavior without biting toward an unfamiliar mouse than control diet-fed SI mice (SI soy: total duration, 33.2 ± 13.4 s, *q*_32_ = 4.330, *p* = 0.0184; mean duration, 6.9 ± 2.6 s, *q*_32_ = 4.355, *p* = 0.0210 *vs*. SI control; Fig. 2C,D), and had no significant difference from the GH groups (total duration, *q*_32_ = 2.660, *p* = 0.2563; mean duration, *q*_32_ = 2.897, *p* = 0.1921 *vs*. GH control and soy; Fig. 2C,D). Two-way ANOVA for effects on the number and total duration of exploration behavior showed significant main effects of diet (number: *F*_(1, 32)_ = 11.23, *p* = 0.0021; total duration: *F*_(1, 32)_ = 14.53, *p* = 0.0006) but no main effects of rearing (number: *F*_(1, 32)_ = 0.1255, *p* = 0.4159; total duration: *F*_(1, 32)_ = 2.475, *p* = 0.1255; Supplementary Fig. S1A,B). For mean exploration duration, there were main effects of rearing (*F*_(1, 32)_ = 20.31, *p* < 0.0001) but no main effects of diet (*F*_(1, 32)_ = 2.680, *p* = 0.1114; Supplementary Fig. S1C). Control diet-fed SI mice had significantly longer mean duration of exploration than GH mice (*p* < 0.001 *vs*. GH control and soy; Supplementary Fig. S1C). Overall, these data indicated that ingestion of soy peptides caused a decrease in aggression induced by isolation rearing.

**Figure 3:**
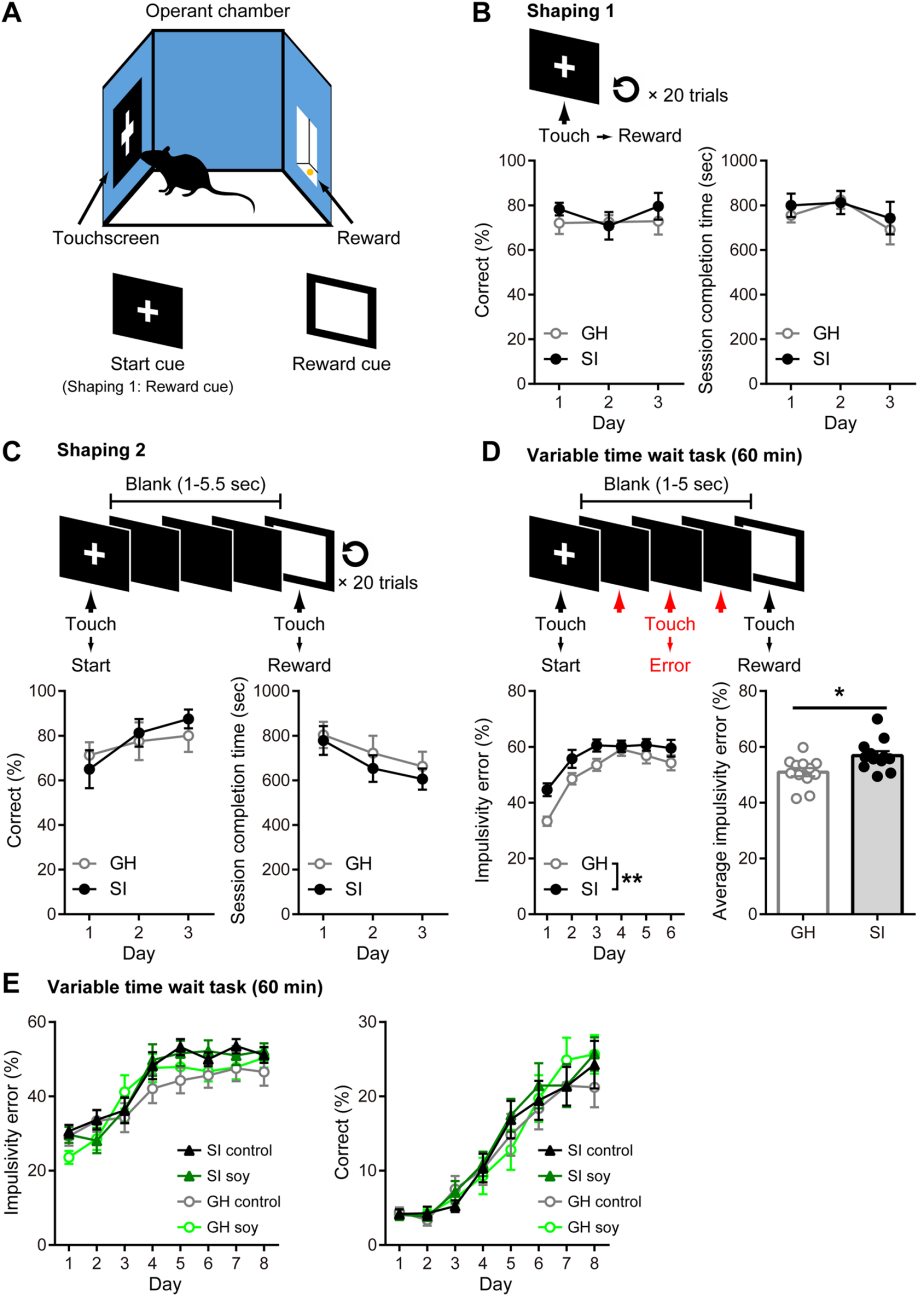
Effects of soy peptides on impulsivity. **(A)** Schematic describing the touchscreen operant chamber and variable time wait task. White cross and square images are used as start cue and reward cue, respectively. Impulsivity response was defined as a response elicited during the blank period. **(B,C)** *Top*, Schematic describing shaping 1 (B) or 2 (**C**). *Bottom*, percentage of the correct response (left) and duration of trials completed (right) is shown for group-housed (GH; open circles; *n* = 12) and socially isolated (SI) mice (closed circles; *n* = 12). **(D)** The percentage of the impulsivity error during daily task (left) and average of percentage of impulsivity error (right) is shown for GH (open circles in left, white bar in right; *n* = 12) and SI mice (closed circles in left, black bar in right; *n* = 12). SI mice were more impulsive across all trails than GH mice (two-way ANOVA; **p < 0.01 vs. GH mice in left). Student’s *t*-test, *p < 0.05 compared with GH mice in right. Data are expressed as mean and SEM. **(E)** During each session in the variable time wait task, the percentage of the impulsivity error (left) and correctly performed task (correct) (right) are shown for SI mice given control diet (black closed triangles, *n* = 6) or soy peptide diet (dark green closed triangles, *n* = 6) and GH mice given control diet (gray open circles, *n* = 6) or soy peptide diet (light green open circles, *n* = 6). SI rearing increased impulsivity error (three-way ANOVA, *F*_(1, 138)_ = 6.743, *p* = 0.00104 for group) independent of diet (three-way ANOVA, *F*_(1, 138)_ = 0.07674, *p* = 0.7822 for group) with no interaction. There are no main effects of diet and rearing on correctly performed task. Data are expressed as mean and SEM.

Next, using the touch panel operant device (Fig. 3A), we evaluated waiting impulsivity, known as anticipatory or premature responding before the appearance of a target, with the variable time wait task. In the shaping procedure, GH and SI mice had similar motivation for the visual cue (white cross) on the touchscreen (Fig. 3B). The correct response rates (*n* = 12, *F*_(1, 11)_ = 1.590, *p* = 0.2334) and the session completion time (*F*_(1, 11)_ = 0.2887, *p* = 0.6017) were similar between the groups across test days (Fig. 3B). There was also no effect of SI on behavior response for two different visual cues: one (white cross) associated with trial-start and another (white square) associated with reward (*n* = 12, *F*_(1, 11)_ = 0.09309, *p* = 0.7660; Fig. 3C). Both groups of mice demonstrated an increase in the correct response rates (*F*_(2, 22)_ = 8.954, *p* = 0.0014) and a decrease in the session completion time (*F*_(2, 22)_ = 13.83, *p* = 0.0001) to a similar extent over test days (Fig. 3C).

However, in the variable time wait task, the rates of impulsive response that occurred before the appearance of the white square image were significantly higher in the SI mice than in the GH mice over test days (*n* = 12, *F*_(1, 11)_ = 11.74, *p* = 0.0057; Fig. 3D) and the average impulsivity error in the SI mice was significantly higher than that in the GH mice (SI: 56.88 ± 1.61%; GH: 50.94 ± 1.48%, *t*_22_ = 2.726, *p* = 0.0123; Fig. 3D). Hence, we examined the effects of ingestion of soy peptides on impulsive behavior in the variable time wait task. As a result, we found that the levels of impulsive response of soy peptide-fed SI mice was similar to that of control diet-fed SI mice across all trials (*n* = 18, *F*_(1, 17)_ = 0.06909, *p* = 0.7958). Three-way ANOVA for effects on impulsive response showed significant main effects of time (*F*_(1, 136)_ = 39.17, *p* < 0.0001) and rearing (*F*_(1, 136)_ = 6.743, *p* = 0.0104); however, there was no main effect of diet (*F*_(1, 136)_ = 0.07674, *p* = 0.7822) nor any interactions with it (*F*_(7, 136)_ = 1.008, *p* = 0.4287; Fig. 3E, left).

Furthermore, the correct response rates of all four groups increased significantly over time (*n* = 18, *F*_(7, 136)_ = 63.87, *p* < 0.0001), with no significant main effect of rearing (*F*_(1, 136)_ = 0.8553, *p* = 0.3567) or diet (*F*_(1, 136)_ = 0.7607, *p* = 0.3846; Fig. 3E, right). These results suggested that ingestion of soy peptides had no significant effect on impulsive behavior.

### Soy peptide ingestion alleviates impairment of fear memory induced by SI

**Figure 4:**
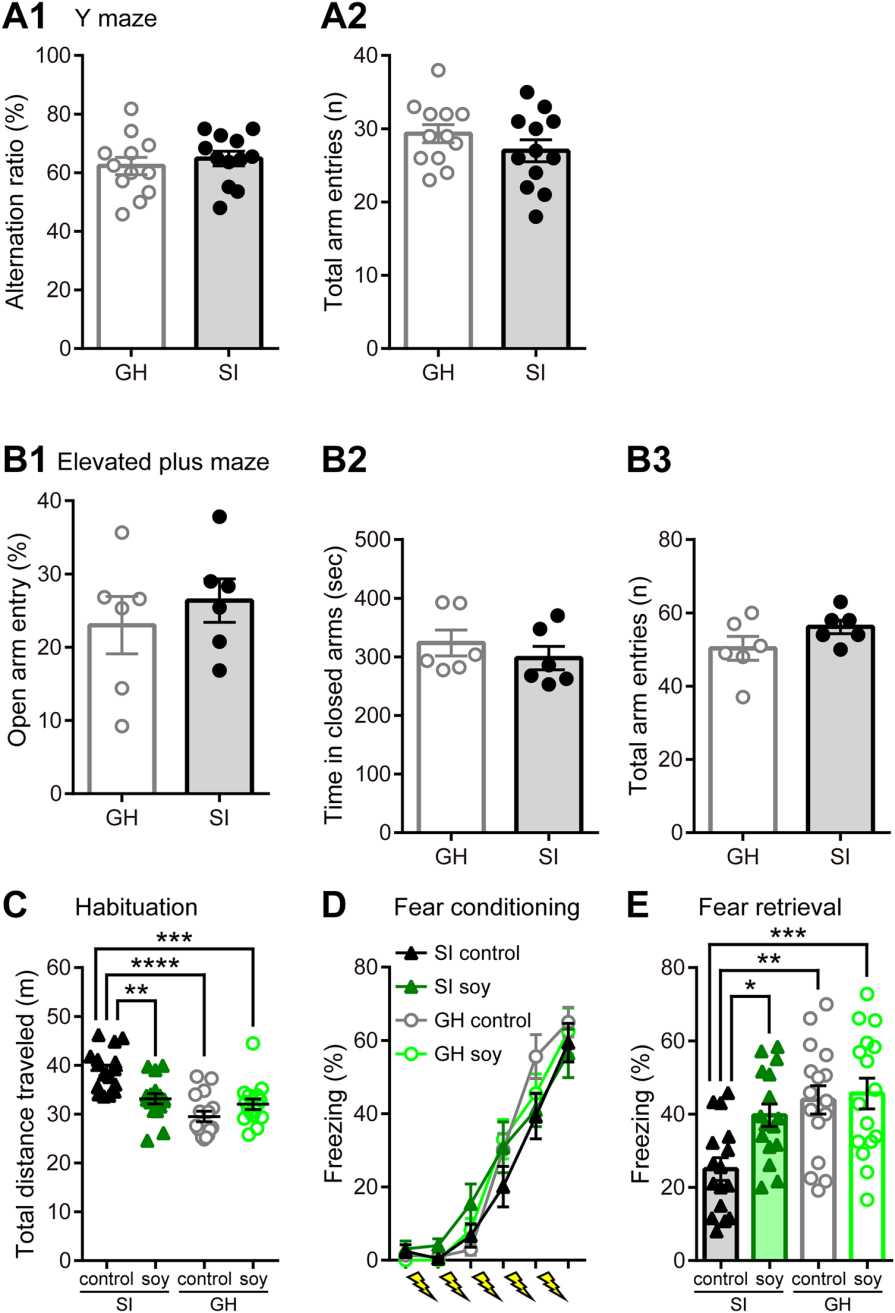
Effects of soy peptides on spatial working memory, anxiety-like behavior, and contextual fear memory. **(A)** The percentage of spontaneous alternation behavior (**A1**) and total number of arm entries (**A2**) during a 6-min Y maze session by group-housed (GH; *n* = 12; white bars) and socially isolated (SI) mice (*n* = 12; black bars). SI mice did not show any difference in behavior when compared with GH mice on the elevated-plus maze and Y maze. **(B)** The percentages of time spent in open arms (**B1**), stay times of closed arms (**B2**), and number of total entries into both open and closed arms (**B3**) during a 10-min elevated-plus maze session by GH (*n* = 6; white bars) and SI mice (*n* = 6; grey bars). **(C)** Total distance of 30-min period for SI mice given control diet (black closed triangles, *n* = 16) or soy peptide diet (dark green closed triangles, *n* = 16) and GH mice given control diet (gray open circles, *n* = 16) or soy peptide diet (light green open circles, *n* = 16) in habituation phase. Hyperactive behavior was observed in SI mice fed with control diet, but not in the other three groups. **(D)** In contextual fear conditioning, the first five data points represent the averaged freezing percentage (%) during 15 s before each electrical shock (reflecting inter-unconditioned stimulus freezing) and the last data points represent freezing (average percent during 15 s period) 15 s after the last shock. There are no significant differences between the groups (*n* = 16). **(E)** Summary data of freezing during contextual fear retrieval 24 h after training. Soy peptide diet-fed SI mice (dark green closed triangles, *n* = 16) exhibited significantly more freezing than control diet-fed SI mice (black closed triangles, *n* = 16), but did not differ significantly from the other GH mice (control diet-fed GH mice: gray open circles, *n* = 16; soy peptide-diet fed GH mice: light green open circles, *n* = 16). Data are expressed as mean and SEM. ^*^p < 0.05, ^**^p < 0.01, ^***^p < 0.001, and ^****^p < 0.0001; two-way ANOVA with Tukey *post*-*hoc* test.

SI mice exhibited no significant differences in spatial working memory performance on the Y maze when compared with GH mice (alternation ratio; GH: 62.30 ± 2.83%, SI: 64.78 ± 2.39%, *n* = 12, *t*_22_ = 0.6422, *p* = 0.5274; Fig. 4A1,A2). Additionally, there was no significant difference in the degree of anxiety-like behavior in the elevated-plus maze between SI and GH mice (open arm entry; GH: 23.01 ± 3.56%, SI: 26.36 ± 2.72%, *n* = 6, *t*_10_ = 0.6823, *p* = 0.5105; Fig. 4B1–3).

Next, we examined the effects of ingestion of soy peptides on fear memory in hippocampus-dependent contextual fear conditioning. During the habituation phase, post-weaning SI led to hyperactive behavior in the novel environment (Fig. 4C). Control diet-fed SI mice showed a significant increase in total distance traveled (SI control: 38.96 ± 1.03 m, *n* = 16, main effect of rearing [*F*_(1, 60)_ = 24.59, *p* < 0.0001], *q*_60_ = 8.875, *p* < 0.0001 *vs*. GH control: 29.52 ± 1.05 m; Fig. 4C). In addition, we found that SI-induced hyperactivity was significantly mitigated by the ingestion of soy peptides (SI soy: 33.13 ± 1.02 m, no main effect of diet [*F*_(1, 60)_ = 2.433, *p* = 0.1241] and a significant interaction between rearing and diet [*F*_(1, 60)_ = 15.34, *p* = 0.0002]; *n* = 16, *q*_60_ = 5.476, *p* = 0.0015 *vs*. SI control; *q*_60_ = 1.042, *p* = 0.8819 *vs*. GH soy: 32.03 ± 1.02 m; Fig. 4C). The percent center time, a measure of anxiety, did not differ significantly among these four groups (SI control: 8.37 ± 0.72%, SI soy: 6.89 ± 0.70%, GH control: 7.79 ± 0.72%, GH soy: 7.49 ± 0.74%, *n* = 16), i.e., no significant main effects of diet (*F*_(1, 60)_ = 1.428, *p* = 0.2367) and rearing (*F*_(1, 60)_ = 0.0001101, *p* = 0.9917; Supplementary Fig. S3A).

On the training day, all four groups showed a significant increase in freezing response during the fear acquisition phase (*n* = 16, *F*_(5, 75)_ = 205.6, *p* < 0.0001), with no significant differences between groups (*F*_(3, 45)_ = 0.5512, *p* = 0.6499; Fig. 4D). However, during contextual testing conducted 24 h after conditioning, there was a significant effect of the diet on the freezing response of SI mice (main effects of diet [*F*_(1, 60)_ = 5.231, *p* = 0.0257] and rearing [*F*_(1, 60)_ = 11.91, *p* = 0.0010]), with the soy peptide-fed SI mice showing more freezing than the control diet-fed SI mice (SI soy: 39.68 ± 2.98%, *n* = 16, *q*_60_ = 4.091, *p* = 0.0266 *vs*. SI control: 24.98 ± 2.97%; Fig. 4E). There was no significant difference between soy peptide- and control diet-fed GH mice (GH soy: 45.59 ± 4.07%; GH control: 43.86 ± 3.76%, *n* = 16; *q*_60_ = 0.4828, *p* = 0.9862; Fig. 4E). To assess whether the changes in locomotor activity affected contextual freezing, we analyzed baseline activity prior to the shock on the day of conditioning. The baseline activity did not differ significantly among these four groups (SI control: 449,489 ± 25,002, SI soy: 420,183 ± 23,163, GH control: 370,255 ± 25,633, GH soy: 45,6017 ± 18,171, *n* = 16), i.e., no significant main effects of diet (*F*_(1, 60)_ = 1.391, *p* = 0.2430) and rearing (*F*_(1, 60)_ = 0.8218, *p* = 0.3683; Supplementary Fig. S3B). Furthermore, during the 2-s shock no differences in shock reactivity were found (SI control: 13,105 ± 399, SI soy: 13,043 ± 349, GH control: 13,143 ± 380, GH soy: 13,119 ± 385, *n* = 16, no significant main effects of diet [*F*_(1, 60)_ = 0.01186, *p* = 0.9136] and rearing [*F*_(1, 60)_ = 0.02099, *p* = 0.8853]; Supplementary Fig. S3C). These data suggest that social isolation-induced impairment of contextual fear memory is alleviated by soy peptides, and this is not due to a change in locomotor activity and shock sensitivity^39^.

### Microbiota composition is altered by soy peptide ingestion

To explore the effect of soy peptides on gut microbial composition in GH and SI mice, we performed bacterial 16S rRNA gene sequencing in the fecal samples of these mice. We found that the composition of the gut microbiota characteristically changed in soy peptide-fed mice, independent of socially-isolated rearing (pink, cyan, and green mark, Supplementary Table S2), while there were differences, although not statistically significant, between GH and SI mice at the species level (Supplementary Fig. S2).

**Figure 5:**
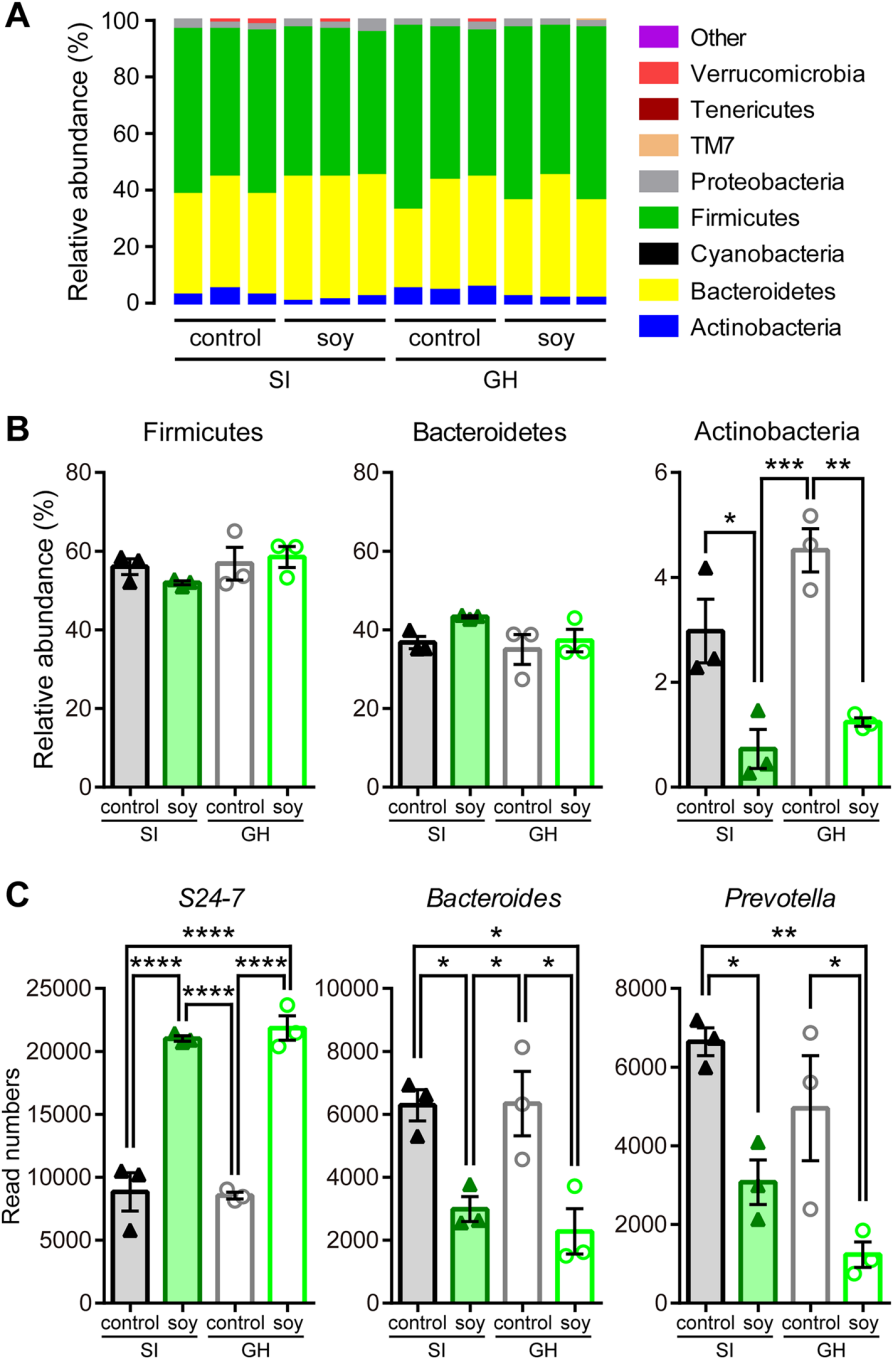
Effects of soy peptides on microbiota composition. **(A)** Relative abundance (%) plot of the most important phyla in socially isolated (SI) and group-housed (GH) mice fed with control or soy peptide diet for a month. **(B**,**C)** Relative abundance (%) of phyla (**B**) and normalized read number of species (**C**) in the gut microbiota of SI mice given control diet (black closed triangles, *n* = 3) or soy peptide diet (dark green closed triangles, *n* = 3) and group-housed (GH) mice given control diet (gray open circles, *n* = 3) or soy peptide diet (light green open circles, *n* = 3). Data are expressed as mean and SEM. *p < 0.05, **p < 0.01, ***p < 0.001, and ****p < 0.0001; two-way ANOVA with Tukey *post-hoc* test.

At the phylum level, no profound differences in the relative abundance of the major bacterial phyla were observed between groups (*n* = 3; Fig. 5A and Supplementary Fig. S1). The relative abundances of *Firmicutes* and *Bacteroidetes*, which accounted for more than 90% of the total gut microbiota, were not significantly different between the groups (*Firmicutes*: *n* = 3, no main effects of diet [*F*_(1, 8)_ = 0.2006, *p* = 0.6661] and rearing [*F*_(1, 8)_ = 1.870, *p* = 0.2086]; *Bacteroidetes*: *n* = 3, no main effects of diet [*F*_(1,8)_ = 3.038, *p* = 0.1195] and rearing [*F*_(1, 8)_ = 2.423, *p* = 0.1582]), although *Actinobacteria* were significantly less abundant in the soy peptide-fed groups than in the control diet-fed groups (*n* = 3, main effects of diet [*F*_(1, 8)_ = 44.68, *p* = 0.0002] and rearing [*F*_(1, 8)_ = 6.175, *p* = 0.0378], and no significant interaction between rearing and diet [*F*_(1, 8)_ = 1.544, *p* = 0.2492]; Fig. 5A,B).

However, at the major species level, the relative abundance of *S24-7*, the most abundant species, was drastically increased (*n* = 3, main effect of diet [*F*_(1, 8)_ = 191.8, *p* < 0.0001]; no main effect of rearing [*F*_(1, 8)_ = 0.08663, *p* = 0.7760]). *Allobaculum* (*n* = 3, main effect of diet [*F*_(1, 8)_ = 12.40, *p* = 0.0078]; no main effect of rearing [*F*_(1, 8)_ = 2.441, *p* = 0.1568]), *Bacteroides* (*n* = 3, main effect of diet [*F*_(1, 8)_ = 27.41, *p* = 0.0008]; no main effect of rearing [*F*_(1, 8)_ = 0.2180, *p* = 0.6531]), and *Prevotella* (*n* = 3, main effects of diet [*F*_(1, 8)_ = 22.80, *p* = 0.0014] and rearing [*F*_(1, 8)_ = 5.348, *p* = 0.0495]) were significantly decreased by the ingestion of soy peptides (Fig. 5C and Supplementary Table S2). These data suggested that soy peptides altered the gut microbiota composition, although juvenile SI had no potent effect.

### Soy peptide ingestion reverses a decrease in synaptic protein expression in the mPFC and phosphorylation of CREB in the hippocampus of SI mice and upregulates phosphorylation of AMPK in the hippocampus

Since the effects of soy peptides on behavioral changes induced by post-weaning chronic SI were not correlated with changes in microbiota, we examined the direct effects of ingestion of soy peptides on neurochemical signaling mechanisms in the brain. In the subcortical emotion-processing regions, including the hypothalamus and amygdala^16,40^ (Supplementary Fig. S4A), there was no change in the mRNA expression of the *Tac1* (GH: 1.00 ± 0.07%, SI: 0.98 ± 0.03%, *n* = 6, *t*_10_ = 0.2029, *p* = 0.8433; Supplementary Fig. S4B) and *Tac2* (CeA: GH, 1.00 ± 0.08%, SI, 1.18 ± 0.253%, *n* = 4, *t*_6_ = 0.6032, *p* = 0.5684; DMH: GH, 1.00 ± 0.07%, SI, 0.95 ± 0.16%, *n* = 6, *t*_10_ = 0.2415, *p* = 0.8172; Supplementary Fig. S4C, D), both of which are components of the signaling pathway associated with aggression after SI; therefore, we focused on higher-order brain regions.

**Figure 6:**
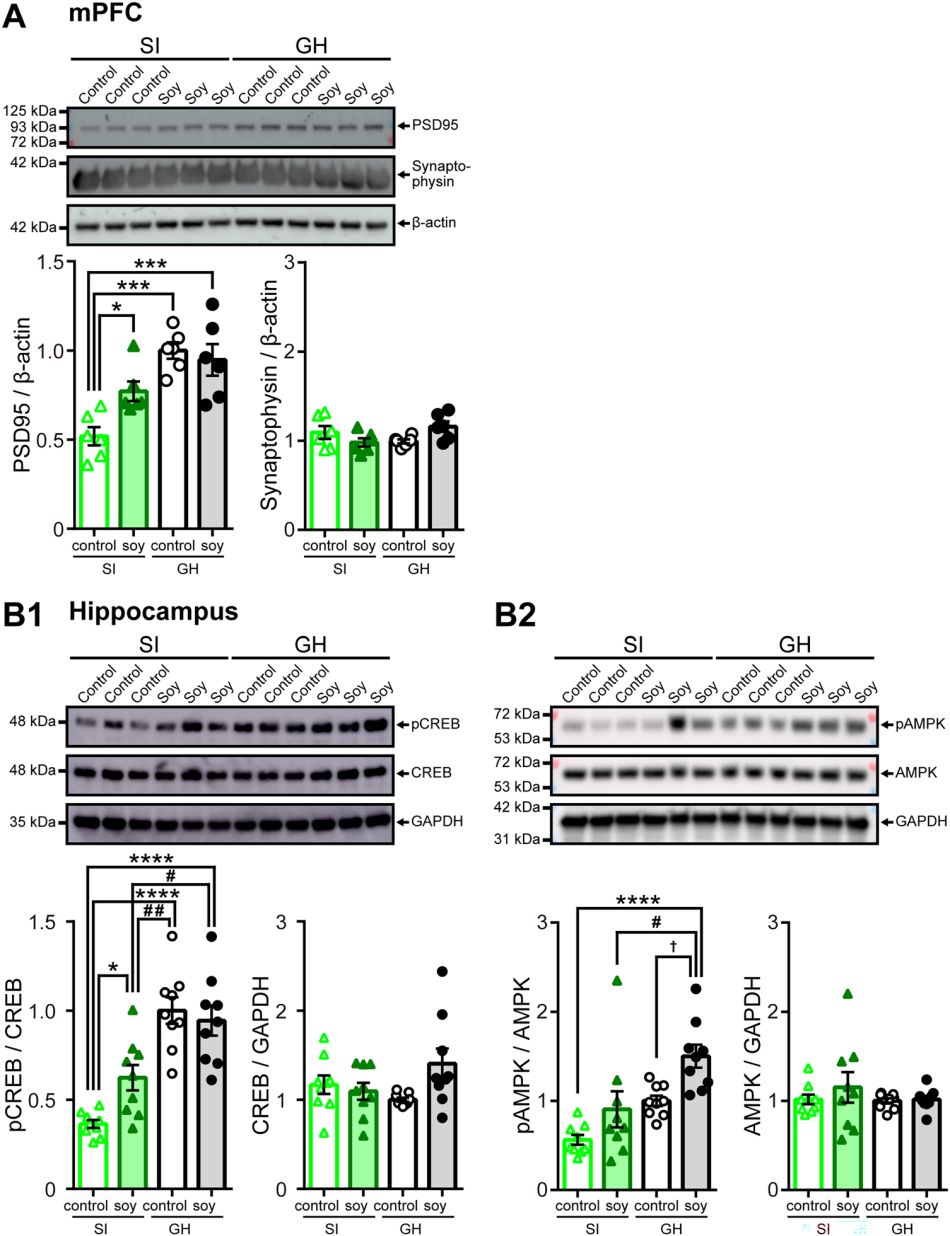
Effects of soy peptides on the expression of synaptic-associated proteins in the medial prefrontal cortex and phosphorylation of CREB and AMPK in the hippocampus. **(A)** *Top*, Representative western blotting images showing the levels of postsynaptic density 95 (PSD95), synaptophysin, and β-actin in the medial prefrontal cortex (mPFC) from SI and GH mice fed with control or soy peptide diet. The original blots are presented in Supplementary Fig. S5. *Bottom*, Quantitative densitometric analysis of western blots. Ratios for PSD95/β-actin (left) and synaptophysin/β-actin (right) of SI mice given control diet (black closed triangles, *n* = 6) or soy peptide diet (dark green closed triangles, *n* = 6) and GH mice given control diet (gray open circles, *n* = 6) or soy peptide diet (light green open circles, *n* = 6) are shown. The fold change was normalized to control diet-fed GH mice. **(B)** *Top*, Representative western blotting images showing the levels of and phosphorylation of CREB (pCREB), total CREB (**B1**), phosphorylation of AMPK (pAMPK), total AMPK (**B2**), and GAPDH (**B1**,**B2**) in the hippocampi from SI and GH mice fed with control or soy peptide diet. The original blots are presented in Supplementary Fig. S6 and 7. *Bottom*, Quantitative densitometric analysis of western blots. Ratios for pCREB/CREB (**B1**, left), CREB/GAPDH (**B1**, right), pAMPK/AMPK (**B2**, left), and AMPK/GAPDH (**B2**, right) of SI mice given control diet (black closed triangles, *n* = 9) or soy peptide diet (dark green closed triangles, *n* = 9) and GH mice given control diet (gray open circles, *n* = 9) or soy peptide diet (light green open circles, *n* = 9) are shown. The fold change was normalized to control diet-fed GH mice. Reduced PSD95 and pCREB protein levels in control diet-fed SI mice are significantly ameliorated in soy peptide diet-fed SI mice. The pAMPK protein level increased by feeding soy peptide diet. β-actin and GAPDH served as the loading control. Data are expressed as mean and SEM. *p < 0.05, ***p < 0.001, and ****p < 0.0001 *vs*. SI control; ^#^*p* < 0.05 and ^##^*p* < 0.01 *vs*. SI soy; ^†^*p* < 0.05 *vs*. GH control; two-way ANOVA with Tukey *post*-*hoc* test.

As social isolation changes the expression of synaptic-associated proteins in the mPFC^20,41^, we measured the levels of PSD95 and synaptophysin in the mPFC via western blotting (Supplementary Fig. S5). ANOVA showed a significant main effect of rearing (*F*_(1, 20)_ = 27.46, *p* < 0.0001), but not of diet (*F*_(1, 20)_ = 2.539, *p* = 0.1268), and a significant effect interaction between rearing and diet (*F*_(1, 20)_ = 5.907, *p* = 0.0246; Fig. 6A). Control diet-fed SI mice showed significantly decreased expression of PSD95 protein in the mPFC than GH mice, both control diet-fed and soy peptide-fed (SI control: 0.52 ± 0.05; *n* = 6; *q*_20_ = 7.671, *p* = 0.0001 *vs*. GH control: 1.00 ± 0.04; *q*_20_ = 6.834, *p* = 0.0005 *vs*. GH soy: 0.95 ± 0.08; Fig. 6A). Contrastingly, this reduction was recovered to nearly control level in soy peptide-fed SI mice (SI soy: 0.77 ± 0.05; *n* = 6; *q*_20_ = 4.024, *p* = 0.0455 *vs*. SI control; *q*_20_ = 2.810, *p* = 0.2259 *vs*. GH soy; Fig. 6A). On the contrary, synaptophysin expression did not demonstrate a change after social isolation and ingestion of soy peptides (*n* = 6, no main effects of diet [*F*_(1, 20)_ = 0.2485, *p* = 0.6236] and rearing [*F*_(1, 20)_ = 0.5062, *p* = 0.4850]; significant interaction [*F*_(1, 20)_ = 6.590, *p* = 0.0184]; Fig. 6A).

Accumulating evidence indicates that CREB plays a critical role in hippocampus-dependent contextual fear memory^42^. Thus, we examined the levels of phosphorylation of CREB (pCERB) in the hippocampus (Supplementary Fig. S6). ANOVA showed a significant main effect of rearing (*F*_(1, 32)_ = 50.54, *p* < 0.0001), but not of diet (*F*_(1, 32)_ = 2.291, *p* = 0.1400), and a significant effect interaction between rearing and diet (*F*_(1, 32)_ = 5.502, *p* = 0.0254; Fig. 6B2). The levels of pCREB were significantly decreased in control diet-fed SI mice (SI control: 0.36 ± 0.02; *n* = 9; *q*_32_ = 9.454, *p* < 0.0001 *vs*. GH control: 1.00 ± 0.07; *q*_32_ = 8.622, *p* < 0.0001 *vs*. GH soy: 0.94 ± 0.08; Fig. 6B1), whereas the levels of pCREB partially recovered in soy peptide-fed mice (SI soy: 0.62 ± 0.07; *n* = 9; *q*_32_ = 9.454, *p* < 0.0001 *vs*. SI control; *q*_32_ = 5.595, *p* = 0.0021 *vs*. GH control; *q*_32_ = 4.763, *p* = 0.0102 *vs*. GH soy; Fig. 6B1). There was no significant difference between the four groups in the expression of total CREB (*n* = 9, no main effects of diet [*F*_(1, 32)_ = 2.255, *p* = 0.1430] and rearing [*F*_(1, 32)_ = 0.4084, *p* = 0.5273]; significant interaction [*F*_(1, 32)_ = 4.706, *p* = 0.0376]; Fig. 6B1).

To investigate the signaling mechanisms involving PSD95 and CREB that are affected by soy peptides supplementation, we examined levels of phosphorylation of AMPK (pAMPK), known as primary target signaling of a component of soy peptides^37^, in the hippocampus (Supplementary Fig. S7). ANOVA showed significant main effects of rearing (*F*_(1, 32)_ = 16.69, *p* = 0.0003) and diet (*F*_(1, 32)_ = 11.18, *p* = 0.0021), and no significant effect interaction between rearing and diet (*F*_(1, 32)_ = 0.3890, *p* = 0.5372; Fig. 6B2). The levels of pAMPK tended to decrease in control diet-fed SI mice (SI control: 0.56 ± 0.05; *n* = 9; *q*_32_ = 3.462, *p* = 0.0883 *vs*. GH control: 1.00 ± 0.05), whereas soy peptide-fed and control diet-fed GH mice showed an almost equal level of pAMPK in the hippocampus (SI soy: 0.91 ± 0.19; *n* = 9; *q*_32_ = 0.7416, *p* = 0.9526 *vs*. GH soy; Fig. 6B2). Interestingly, soy diet-fed GH mice showed significantly increased levels of pAMPK than the other groups (GH soy: 1.50 ± 0.12; *n* = 9; *q*_32_ = 7.429, *p* < 0.0001 *vs*. SI control; *q*_32_ = 4.709, *p* = 0.0112 *vs*. SI soy; *q*_32_ = 3.967, *p* = 0.0402 *vs*. GH control; Fig. 6B2), conforming that soy peptides have an agonistic effect on AMPK signaling in vivo. There was no significant difference between the four groups in the expression of total AMPK (*n* = 9, no main effects of diet [*F*_(1, 32)_ = 0.6577, *p* = 0.4234] and rearing [*F*_(1, 32)_ = 0.6849, *p* = 0.4140]; Fig. 6B2).

Together, these results suggested that soy peptides ameliorated the social isolation-induced reduction in PSD95 level in the mPFC and CREB activation in the hippocampus and increased hippocampal pAMPK levels.

## Discussion

In this study, we investigated whether ingestion of soy peptides mitigates detrimental alterations in brain function induced by post-weaning SI. We found that soy peptides effectively reduced aggressive behavior, hyperactivity, and fear memory impairment, accompanied by restoration of decrease in both PSD95 levels of the mPFC and phosphorylation of hippocampal CREB by SI. We also observed that hippocampal pAMPK levels and microbiota composition were altered by the ingestion of soy peptides.

To our knowledge, this study is the first to demonstrate that ingestion of soy peptides attenuates the adverse behavioral effects of juvenile SI in mice. Mice isolated from weaning for 4 weeks exhibited attacking behaviors toward an unfamiliar mouse, including clinching and biting, high impulsivity in the variable time wait task, increased motility in the novel environment, and impairment of contextual fear memory. These behavioral abnormalities, except for impulsivity, were alleviated by ingestion of soy peptides during SI-rearing, suggesting that soy peptides have significant mitigative effects on aggressive behavior, hyper-locomotion activity, and fear memory deficit resulting from social isolation stress exposure during early life.

In our study, we showed that the microbiota composition was strongly influenced by ingestion of soy peptides, independent of rearing conditions. Hence, although changes in the gut microbial environment cannot explain the mitigating effect of soy peptides on SI-induced behavioral deficits, a growing body of evidence indicates that microbiota influences brain function via metabolic, endocrine, immune, and neural pathways^38^. For example, transplantation of microbiota shaped by a high-fat diet impairs cognitive-behavioral performance by increased inflammation^43^. Treatment with a probiotic *Lactobacillus* species during chronic stress exposure improves despair behaviors by modulating kynurenine metabolism^44^, indicating stress recovery via gut microbes. However, since at least some dipeptides in soy peptides can reach the brain parenchyma across the blood–brain barrier, the possibility of direct modulation of neuronal signaling by soy peptides cannot be excluded.

Previous studies have been shown that aggression is one of the major characteristics of juvenile SI, especially under social interaction with a novel same-sex conspecific^45^, and correspondingly, is a common feature of several psychiatric disorders^46^. Thus, stressed mice easily turn social contacts into attack behaviors. However, soy peptides-fed male SI mice showed reduced aggression toward unfamiliar male mice, as indicated by prolonged exploratory behaviors and less aggressive behaviors without attacking. Therefore, soy peptides may exert a protective effect against SI stress, which is consistent with the results from previous studies in human volunteers^27,28^. Aggression is modulated by canonical neurotransmitters such as serotonin, dopamine, noradrenaline, and GABA^47^, all of which are affected in discrete brain regions, particularly in the mPFC, by SI^48,49^. Although the contribution of soy peptides to the synthesis and turnover of catecholamines and amino acids in the brain have been reported^35,50^, their effects on the brain in psychiatric disease are unclear. Consequently, elucidating the specific mechanisms underlying the roles of soy peptides in neurotransmitter systems, which are associated with emotional behaviors, is an important topic for future research.

The mPFC is involved in the inhibitory control of emotional behaviors, and disruptions of this control results in emotional outbursts, including high aggression and impulsivity. Since synaptic networks of the frontal lobe are dynamically reorganized by experience, with a gradual increase in the expression of the synaptic-associated proteins, including PSD95, during the juvenile and adolescent ages^51^, social deprivation from weaning disturbs the functional maturation of synapses in the mPFC^52^. Mice reared in SI exhibit reductions in dendritic spine density, synaptic excitability, and synaptic proteins in the frontal brain regions, thereby altering their behaviors in adulthood^20,22,41^. Interestingly, amelioration of these synaptic abnormalities suppresses stress-induced aggression outbursts and depression-like behavior in SI mice^53,54^. Therefore, soy peptide intake may contribute to synaptic maturation, which in turn may reduce SI-induced aggression and hyperactivity.

Meanwhile, we cannot exclude the possibility that soy peptides affect downstream brain regions that directly control aggression. However, in the present study, we observed that the expression of neuropeptides tachykinin, a key mediator of stress responses including aggression, in the hypothalamus and amygdala was not altered by SI. The brain tachykinin 2 is upregulated by 2 weeks SI during adulthood from the age of 8–16 weeks as shown by a previous study^16^, but not by 4 weeks SI post-weaning in the current study. The different timing of SI may lead to different degrees of effects on the expression of tachykinin 2^55^. However, given that a previous optogenetic study has also shown that specific activation of the mPFC is enough to suppress aggressive bursts^56^, synaptic modulation by soy peptides might be involved in top-down emotional control processes^25^.

Juvenile SI is known to impair hippocampal synaptic plasticity^57^, which is an important mechanism for long-term memory formation. The present biochemical analysis showed that the amount of pCREB, a key transcription factor for the regulation of memory, was considerably decreased in the hippocampi of SI mice. Neurons with high CREB expression are more likely to be recruited into fear memory trace^58^, which is the physical substrate of memory in the brain. On the contrary, neurons with reduced CREB level are excluded from fear memory allocation, resulting in impairments in memory^58^. Increase in CREB function in the hippocampus is sufficient to revert both anatomical abnormalities and memory deficits in mouse models of Alzheimer's disease, independent of changes in β-amyloid levels or plaque load^59^. An important finding reported here is that soy peptide ingestion alleviates not only reduced CREB activity, but also fear memory deficit in SI mice. We therefore propose that soy peptides may help mediate the formation of fear memory trace by reducing deterioration of CREB signaling related to synaptic plasticity. However, we cannot rule out the possibility that impaired fear memory is mitigated by an additional system other than the actuation of hippocampal CREB signaling, because pCREB levels of soy peptide-fed SI mice were significantly lower than control mice.

We observed that PSD95 upregulation and CREB activation were induced in the mPFC and hippocampus of soy peptide-fed SI mice, respectively. Although the molecular mechanism underlying this regulation remains unknown, the brain–transportable soy peptide, Tyr-Pro, can directly interact with adiponectin receptor 1 (AdipoR1), which is expressed in the brain, as an agonist^37,60^. AdipoR1 has been reported to contribute to the expression of the PSD95 and the activation of CREB^61^, indicating that AdipoR1 signaling could be involved in antistress-like effects by soy peptides. Indeed, chronic ingestion of soy peptides increased the levels of hippocampal pAMPK, which is activated by AdipoR1^62^. However, in contrast to previous results using chronic restraint stress^63^, no significant effect of SI rearing on pAMPK, was observed, indicating that changes in AMPK activity are dependent on the type of stress. Determining the target proteins of soy peptides is crucial to elucidating the signaling pathways responsible for the attenuation of behavioral abnormalities by soy peptides.

There were some limitations to this study. First, the minimum intake of soy peptides that is effective in mitigating behavioral abnormalities has not been determined. Second, the effects of soy peptides in female mice have not been examined. Third, this study did not provide the causal relationship between behavioral changes induced by soy peptides and their underlying molecular mechanisms. Identifying the effective component included in soy peptides that has antistress-like effects should be a focus of future studies.

In conclusion, our findings showed that soy peptides attenuate aggression, hyperactivity, and cognitive dysfunction in mice by mitigating neuronal abnormalities and also affect the microbiota composition. Accumulating evidence indicates that long-term dietary supplementation has beneficial effects on cellular functions and is nearly as effective as medication for mental health without adverse effects^64^. Considering that soy peptides have a mitigative effect on age-related cognitive decline by modulating synaptic plasticity-associated proteins^34^, daily intake of soy peptides may be useful in mitigating progressive deterioration of synaptic function caused by aging and juvenile chronic stress.

## Methods

### Animals and experimental schedule

A total of 319 male C57BL/6J mice (Japan SLC, Inc., Shizuoka, Japan) were used for experiments while a total of 12 male BALB/c mice (7 weeks; Japan SLC, Inc.) were used as unfamiliar mice for dyadic social interaction testing. Animals were maintained under a 12-h light/dark cycle at 24 ± 1 °C and 50 ± 5% relative humidity. The experimental schedule is shown in Fig. 1. After weaning at postnatal day (PND) 21, mice were randomly divided two groups and were group-housed (3 mice/cage) in standard plastic cages (W182 × D260 × H128 mm) or individually housed (socially isolated) in the same size cages until euthanization. They were fed either a control diet, containing 14% casein, or a soy peptide diet, containing 7% casein plus 7% soy peptides (Fuji Oil Co., Ltd., Osaka, Japan) (Supplementary Table S3). The experimental diets and water were provided ad libitum. The daily food consumption was determined by weighing the diets daily for 4 weeks.

At 4 weeks after initiation of SI, behavioral and biological experiments were conducted. Different mice were used in each experiment. For microbial community analysis, fecal samples were collected at 4 weeks after SI. For biological analysis of brain tissue, mice were euthanized by cervical dislocation after anesthesia with 3.5% isoflurane (Wako Pure Chemical Industries, Osaka, Japan) on PND49 outside the housing rooms and then decapitated with scissors. The experimental protocols were approved by the animal experiment ethics committee of Hoshi University and performed in accordance with the Hoshi University guidelines for the care and use of laboratory animals and with the ARRIVE guidelines.

### Dyadic social interaction test

The modified dyadic social interaction test^65^ was used to analyze aggressive behavior 4 weeks after the initiation of SI. Each subject mouse was transferred to a neutral cage identical to the home cage and habituated for 2 min. Age-matched BALB/c unfamiliar mice were individually introduced into the experimental cage for 10 min. Their behavior was video-recorded, and a blinded observer performed ethological analysis of aggression. The behavioral phenotypes of mice were categorized into one of the following three categories, according to the degree of aggressive behaviors: *exploration* (natural social exploratory behavior including facial and anogenital sniffing), *dominance* (social dominant agonistic behavior including mounting and chasing), and *attack* (clinch fights and sustained biting attack).

### Touch panel operant test

#### Apparatus

The touch panel operant device (O’HARA & Co., Ltd., Tokyo, Japan) consists of a trapezoid chamber (W55 × W205 × D135 × H200 mm), an infrared photosensor-embedded 15-inch touch panel, a pellet dispenser that supplies sucrose-containing reward pellets (10 mg; AIN-76A, Research diets, Inc., New Brunswick, NJ, USA) placed on the opposite side of the touch panel, and two water bottles placed on both sides of the chamber in a sound-attenuated box.

#### Habituation

Prior to experiments, the body weights of mice were reduced to 90% of their ad libitum feeding weight via a 2-day dietary restriction. The mice were then acclimated to the reward pellets by providing 10 pellets per mouse in the home cage for 2 days. The next day, they were habituated to the touch panel operant chamber for 1 h and eating the pellets from the reward magazine.

#### Shaping

The operant shaping consisted of two stages: (i) a “white cross” picture was presented on the screen, and reward pellets were delivered when a mouse touched the screen within 60 s; (ii) a session was initiated with the mouse touching the screen while the white cross picture appeared. After a blank period of 1–5.5 s, a “white square” picture was randomly presented on the screen, and reward pellets were delivered when a mouse touched the screen within 60 s. A trial was omitted if a mouse did not touch the screen while the picture was displayed. Each shaping stage was ended after the completion of 20 trials and continued for 3 days.

#### Variable time wait task

Following habituation, the mice underwent a variable time wait task. The procedures were identical to those of *shaping 2*. However, if a mouse touched the screen during the variable blank period, no pellet was rewarded, and the response was recorded as an impulsivity error. This task was terminated after 60 min and continued for 8 days.

### Y maze

The Y-maze apparatus (O’HARA & CO., Ltd.) consists of three identical arms (W30 × D400 × H120 mm each) that are positioned at equal angles (120°). Each mouse was placed at the end of one arm and allowed to explore the maze freely for 6 min. The number and sequence of arm entries were recorded with a video camera. Alternation was defined as three consecutive entries in three different arms on overlapping triplet sets and was measured automatically using the O'HARA's original Y-maze analysis TimeYM2 software (ver.1; https://ohara-time.co.jp/products/y-maze/; O’HARA & Co., Ltd.). The percentage of spontaneous alternation was calculated with the following equation: [(number of alternations)/(total number of arm entries) − 2] × 100.

### Elevated plus maze

The elevated plus maze apparatus (O’HARA & CO., Ltd.) consists of two open arms (W50 × D250 mm each) and two enclosed arms (W50 × D250 × H150 mm each) with a quadrangular central platform (50 × 50 mm) that are elevated 50 cm from the ground. Each mouse was placed on the enclosed arm and allowed to explore the maze freely for 10 min. The number of arm entries and the time spent in the open and closed arms were recorded with a video camera and were measured automatically using the O'HARA's original elevated plus maze analysis TimeEP2 software (ver.1; https://ohara-time.co.jp/products/elevated-plus-maze/; O’HARA & Co., Ltd.).

### Contextual fear conditioning

Four weeks after the initiation of SI, a contextual fear conditioning test was conducted. The conditioning chamber consists of a clear plastic box with a stainless-steel grid floor (W160 × D140 × H120 mm; O’HARA & CO., Ltd.) and was placed in a sound-attenuated box. In assessing exploratory behavior for adaptation to the context, the mice were allowed to explore the conditioning chamber freely for 30 min, and their locomotor activity was measured by the total distance traveled. Time spent in a 48 × 42 mm rectangle region in the center of the chamber was also measured. For training, the mice were placed into a conditioning chamber and exposed to electric foot shocks after 2 min (0.3 mA, five times, 1 s each, separated by 30-s inter-stimulus interval). At 60 s after the last shock, the mice were returned to their home cages. After 24 h, the mice were placed back into the conditioning chamber for 3 min, and their freezing behavior, which was defined as movement that had < 20-pixel changes in 2 s, was measured automatically using the O'HARA's original fear conditioning analysis TimeFZ2 software (ver.1; https://ohara-time.co.jp/products/contextual-and-cued-fear-conditioning-test/; O’HARA & Co., Ltd.). Baseline activity during the 2-min period before the shock and mean shock reactivity during the five presentation of the 1-s shock were also calculated as the cumulative area of motility (pixel size) per 0.5 s.

### 16S ribosomal (r) ribonucleic acid (RNA) gene microbial community analysis

Mouse fecal samples were collected directly into tubes from which microbial deoxyribonucleic acid (DNA) were extracted. Next-generation sequencing of 16S rRNA of the extracted DNA was performed by Repertoire Genesis Inc. (Osaka, Japan). Briefly, the V3-V4 regions of 16S rRNA genes were amplified using polymerase chain reaction (PCR) with individually barcoded universal primers and sequenced by MiSeq next-generation sequencer (Illumina Inc., San Diego, CA, USA) with an average depth of 75 000 reads per sample. The sequence data were analyzed using Flora Genesis original software (ver.20161108; https://www.repertoire.co.jp/en/research/technology/16srrnainfo/; Repertoire Genesis, Inc.).

### Quantitative real-time PCR

Tissue punches were obtained from the medial and central amygdala and dorsomedial hypothalamus and frozen for subsequent processing. Total RNA was extracted and purified using RNeasy Mini Kit (Qiagen, Hilden, Germany) following the manufacture’s protocol. RNA from each sample was reverse transcribed using the PrimeScript™ RT Master Mix kit (Takara Bio Inc., Shiga, Japan). Quantitative real-time PCR was performed to determine relative gene-expression levels with TB Green™ Premix Ex Taq™ II (Tli RNaseH Plus; Takara Bio Inc.), using a StepOne Plus Real-Time PCR (Applied Biosystems, Foster City, CA, USA). All samples were run in triplicate.

The primer sequences were as follows: *Tac1* (forward, 5′- GAT GAA GGA GCT GTC CAA GC -3′; reverse, 5′- TCA CGA AAC AGG AAA CAT GC -3′), *Tac2* (forward, 5′- GCC ATG CTG TTT GCG GCT G -3′; reverse, 5′- CCT TGC TCA GCA CTT TCA GC -3′), and glyceraldehyde-3-phosphate dehydrogenase (*Gapdh)* (forward, 5′- CAT GGC CTT CCG TGT TCC TA -3′; reverse, 5′- GAT GCC TGC TTC ACC ACC TT -3′). *Gapdh* expression was used as an internal control for detection of messenger RNA expression level. Fold changes relative to the control were obtained by using the 2^−ΔΔCt^ method.

### In-situ hybridization

The mice brains were removed, fixed overnight in 4% paraformaldehyde solution, and cryoprotected in 30% sucrose solution for two nights. The fluorescence *in-situ* hybridization was performed on cryostat slices (30 µm) by previously described methods^66^. Briefly, the slices were hybridized using fluorescein isothiocyanate (FITC)-labeled *Tac2* riboprobe (0.3 µg/mL; #RP_050725_03_B09, Allen Brain Atlas probe). The hybridization signals were amplified using anti-FITC antibody conjugated with horseradish peroxidase (1:5,000; Roche Diagnostics, Mannheim, Germany) and TSA Plus DNP kit reagents (NEL701A001KT, PerkinElmer, Waltham, MA, USA). Subsequently, the sections were incubated with anti-DNP antibody conjugated with Alexa 488 (1:500; Molecular Probes, Eugene, OR, USA) to detect the FITC-labeled probe. The sections were mounted using CC/Mount (Diagnostic BioSystems, Pleasanton, CA, USA) and observed using a confocal microscope (FV1200; Olympus, Tokyo, Japan).

### Western blot analysis

The mPFC samples were homogenized in ice-cold Syn-PER™ Synaptic Protein Extraction Reagent (Thermo Fisher Scientific, Waltham, MA, USA) containing protease inhibitor cocktail (P8340; Sigma-Aldrich, St. Louis, MO, USA) with a Dounce-type glass-glass homogenizer and then centrifuged at 1,200 × *g* for 10 min at 4 °C. The supernatants were centrifuged again at 15,000 × *g* for 20 min at 4 °C to pellet the synaptosomal fraction, which was then lysed in lysis buffer (10 mM Tris–HCl, pH 7.5, 2% sodium dodecyl sulfate [SDS], and 1 mM EDTA).

The isolated hippocampi were suspended in ice-cold RIPA buffer (50 mM Tris–HCl, pH 7.5, 150 mM NaCl, 0.5% sodium deoxycholate, 1% Triton X-100, 0.15% SDS, 1 mM sodium orthovanadate, 10 mM NaF, and 1% protease inhibitor cocktail) and homogenized with TissueLyserII (Qiagen). The lysates were centrifuged at 14,000 × *g* to remove debris, and the supernatants were collected. The protein concentrations of the synaptosomal lysate of the mPFC and hippocampal total cell lysate were measured using the BCA protein assay kit (Thermo Fisher Scientific). Equal amounts of protein were separated on SDS polyacrylamide gels and transferred to polyvinylidene difluoride membranes. The membranes were blocked with 5% skimmed milk in Tris-buffered saline with 0.1% Tween-20 for 30 min, and incubated with mouse monoclonal anti-PSD95 (#610495; 1:500, BD Biosciences, Franklin Lakes, NJ, USA), rabbit monoclonal anti-synaptophysin (#ab32127; 1:70,000, Abcam, Cambridge, UK), rabbit polyclonal anti-CREB (#9197; 1:1,000, Cell Signaling Technology), and rabbit polyclonal anti-pAMPK (#2535; 1:1,000, Cell Signaling Technology, Danvers, MA, USA) antibodies overnight at 4 °C. Next, the blots were developed using an HRP-conjugated donkey anti-mouse and rabbit IgG antibody (1:20,000; Jackson ImmunoResearch Laboratories, West Grove, PA, USA) and then the chemiluminescent reagent (Immobilon Western; Millipore, Billerica, MA, USA). The blots were stripped with 62.5 mM Tris–HCl (pH 6.8) containing 100 mM 2-mercaptoethanol and 2% SDS at 60 °C for 30 min, and rehybridized with mouse monoclonal anti-β-actin (#A1978; 1:2,000, Sigma-Aldrich), rabbit polyclonal anti-pCREB (#9198; 1:3,000, Cell Signaling Technology), rabbit polyclonal anti-AMPK (#5831; 1:1,000, Cell Signaling Technology), and rabbit polyclonal anti-GAPDH (#G9545; 1:5,000, Sigma-Aldrich) antibodies for 1 h at room temperature. Quantification of band densities was measured using ImageJ software (National Institutes of Health, Bethesda, MD, USA), and pCREB and pAMPK band densities were normalized to the density of the total CREB and AMPK band, respectively. β-actin and GAPDH were used as loading and normalization control of synaptosomal fraction and total cell lysates, respectively.

### Statistical analysis

All data are presented as mean ± standard error of the mean and were analyzed using GraphPad Prism software (ver.10; https://www.graphpad.com/; GraphPad, La Jolla, CA, USA). For the dyadic social interaction test, differences in behavioral phenotypes were assessed using Fisher’s tests. For other experiments, Student’s* t*-test for statistical comparison of differences between two groups and one-way, two-way, three-way analysis of variance (ANOVA), followed by Tukey’s test for differences between more than two groups. We did not predetermine sample sizes by statistical methods, but our sample sizes were similar to those reported in previous studies in the field^24,34,67^. Differences with *p* < 0.05 were considered significant. The results of all statistical analyzes, including 95% confidence intervals and effect size (η2), are provided in Supplementary Table S4.

### Supplementary Information

Supplementary Information 1.

Supplementary Information 2.

Supplementary Information 3.

## Data Availability

The datasets and 16S rRNA sequencing data generated and analyzed during the current study are available at the Gene Expression Omnibus (GEO) database (Accession Number GSE231557, Token Number gnuduaykfjwdbcz, https://www.ncbi.nlm.nih.gov/geo/) and in the G-Node Infrastructure (GIN) repository, 10.12751/g-node.v7wkqe/, and https://gin.g-node.org/h-tamura/Tamura_Dataset-2023.
