# Supplementary material for: Chronic ingestion of soy peptide supplementation reduces aggressive behavior and abnormal fear memory caused by juvenile social isolation
Source: Sci Rep. 2024 May 21;14:11557. doi: 10.1038/s41598-024-62534-w (PMC11109177; doi:10.1038/s41598-024-62534-w)
Supplement: Supplementary file 1 — Supplementary Information 1. [file 41598_2024_62534_MOESM1_ESM.docx]

**Chronic ingestion of soy peptide supplementation reduces aggressive behavior and abnormal fear memory caused by juvenile social isolation**

Hideki Tamura^1,2*^Akiko Miyazaki^3^, Takashi Kawamura^3^, Hikaru Gotoh^3^, Naoki Yamamoto^4^, Minoru Narita^2,3,5^

^1^Laboratory of Biofunctional Science, Hoshi University School of Pharmacy and Pharmaceutical Sciences, Tokyo, Japan

^2^Institute for Advanced Life Sciences, Hoshi University School of Pharmacy and Pharmaceutical Sciences, Tokyo, Japan

^3^Department of Pharmacology, Hoshi University School of Pharmacy and Pharmaceutical Sciences, Tokyo, Japan

^4^Department of Psychiatry, University of Texas Southwestern Medical Center, Dallas, TX, United States

^5^Department of Pharmacy, National Cancer Center Hospital, Tokyo, Japan

^*^**Corresponding author**: Hideki Tamura

*Tel.: +81-3-5498-5845; E-mail:* [*h-tamura@hoshi.ac.jp*](mailto:h-tamura@hoshi.ac.jp)

# Supplementary Tables:

# Table S1. Body weight and food consumption of SI and GH mice fed with control or soy peptide diet.

|  | **SI** | | **GH** | |  |
| --- | --- | --- | --- | --- | --- |
|  | **Control** | **Soy peptides** | **Control** | **Soy peptides** | ***p* value** |
| **Initial body weight (g)** | 11.28 ± 0.26 | 11.50 ± 0.64 | 11.50 ± 0.62 | 11.87 ± 0.36 | 0.8933 |
| **Body weight at 4 weeks (g)** | 23.61 ± 0.40 | 23.41 ± 0.19 | 23.31 ± 0.31 | 23.59 ± 0.24 | 0.9024 |
| **Body weight gain (g)** | 12.33 ± 0.61 | 11.91 ± 0.56 | 11.81 ± 0.68 | 11.72 ± 0.48 | 0.9117 |
| **Daily food consumption**  **(g/day)** | 3.29 ± 0.06 | 3.19 ± 0.01 | 3.22 ± 0.007 | 3.23 ± 0.01 | 0.2457 |

Body weights of socially isolated (SI) and group-housed (GH) mice before and 4 weeks after being fed a control diet or a soy peptide diet, and the average daily food consumption of each group over the 4 weeks have been presented. The weight gain and daily food consumption are not significantly different among the groups (Weight gain: one-way ANOVA; *F*_(3,20)_ = 0.1756, *p* = 0.9117; Food consumption: *F*_(3,20)_ = 1.497, *p* = 0.2457). Values are presented as means ± SEM. *N* = 6 per group.

# Table S2. Composition of the most abundant gut microbiota at the species level (Excel file).

The numbers of the top 30 abundant bacterial species in socially isolated (SI) and group-housed (GH) mice fed with control or soy peptide diet for a month have been presented. Pink marks indicate significant differences between control and soy peptide diets both in GH and SI mice. Cyan marks indicate significant differences between control and soy peptide diets in GH mice. Green marks indicate significant differences between control and soy peptide diets in SI mice.

# Table S3. Experimental diet composition.

| Composition | Relative amount (%)  control diet soy peptides diet |
| --- | --- |
| Cornstarch  Casein  Soy peptides  Sucrose  Cellulose  Soybean oil  AIN-93 mineral mixture  AIN-93 vitamin mixture  α-cornstarch  Choline bitartrate  _L_-Cystine  *tert*-Butylhydroquinone | 61.0692 61.0692  14 7.0  ― 7.0  10 10  5.0 5.0  4.0 4.0  3.5 3.5  1.0 1.0  1.0 1.0  0.25 0.25  0.18 0.18  0.0008 0.0008 |

# Table S4. Statistical analysis (Excel file).

#
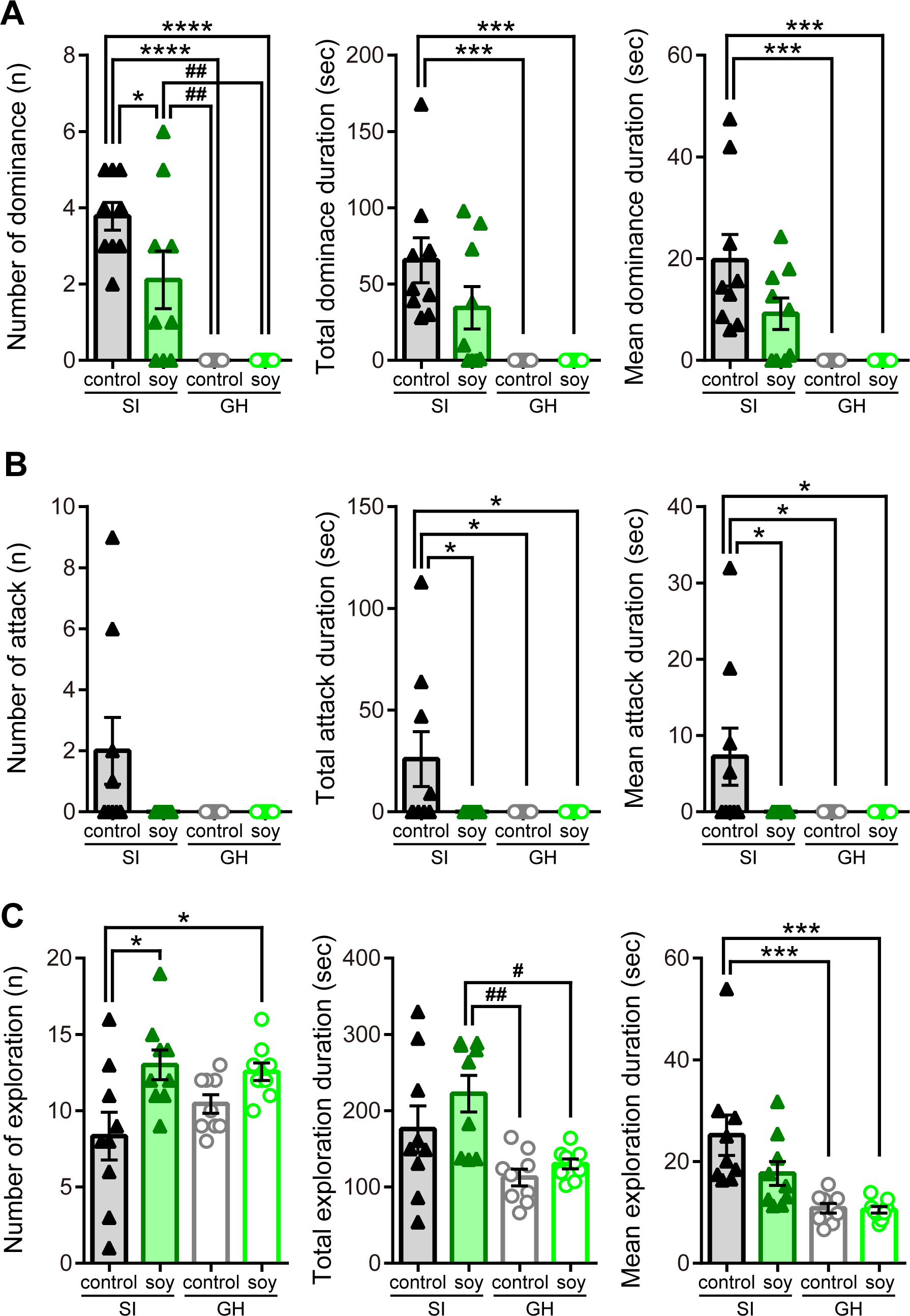
Supplementary Figures:

**Figure S1.** Effects of soy peptides on dominance, attack, and exploration behavior. **(A-C)** Dominance (A), attack (B), and exploration behavior (C) in the dyadic social interaction test are shown for socially isolated (SI) mice given control diet (black closed triangles; *n*=9) or soy peptide diet (dark green closed triangles; *n*=9) and group-housed (GH) mice given control diet (gray open circles; *n*=9) or soy peptide diet (light green open circles; *n*=9). Data are expressed as mean and SEM. ^*^*p*<0.05, ^***^*p*<0.001, and ^****^*p*<0.0001 *vs*. SI control; ^#^*p*<0.05 and ^##^*p*<0.01 *vs*. SI soy; two-way ANOVA with Tukey *post*-*hoc* test.


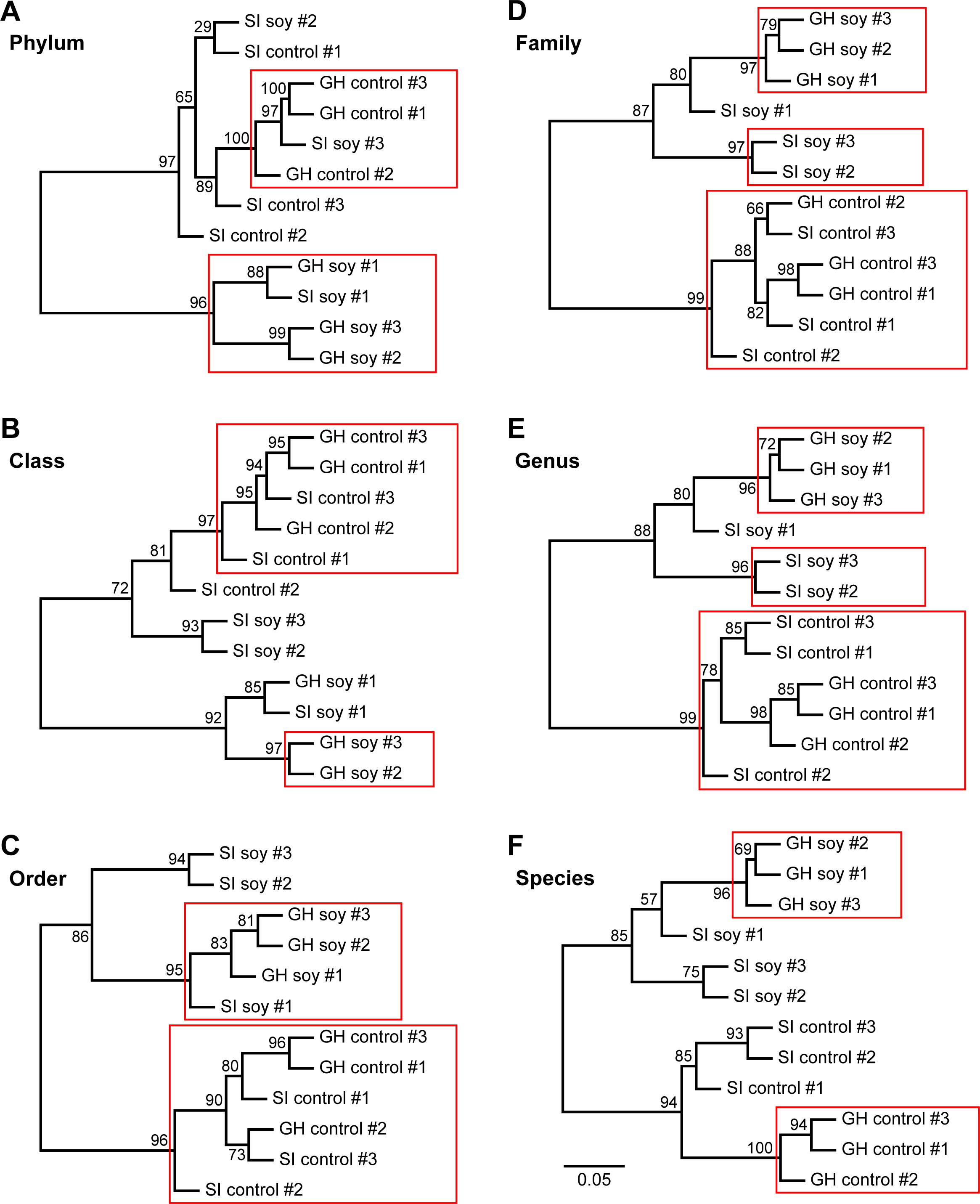


**Figure S2.** Hierarchical clustering tree on each classification level.

Unsupervised hierarchical clustering of relative abundance of gut microbiota in socially isolated (SI) and group-housed (GH) mice fed with control or soy peptide diets for a month (*n* = 3). Values at branches are approximately unbiased (AU) *p* values (%) computed via bootstrap resampling. Clusters with AU ≥ 95 % are indicated by red rectangles.

**
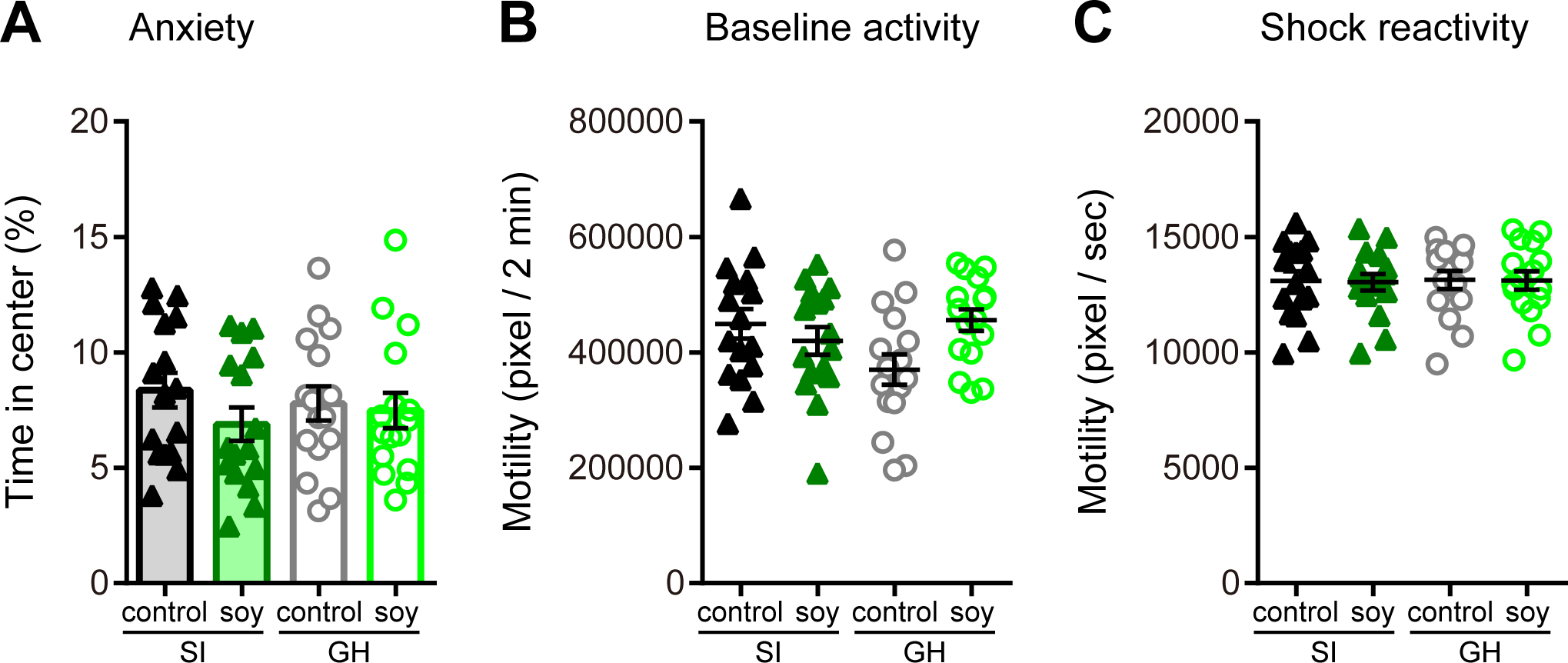
**

**Figure S3.** Impact of anxiety, baseline activity, and shock reactivity.

**(A)** Anxiety-like behavior. Time in center of 30-min period in habituation phase. **(B)** Baseline activity. Motility during the 2-min before the first shock on the day of conditioning. **(C)** Shock reactivity. Average movement during the five 1-sec shock. Anxiety-like behavior, baseline activity before the shock, and shock sensitivity are similar in SI mice given control diet (black closed triangles, n=16) or soy peptide diet (dark green closed triangles, n=16) and GH mice given control diet (gray open circles, n=16) or soy peptide diet (light green open circles, n=16). Data are expressed as mean and SEM.


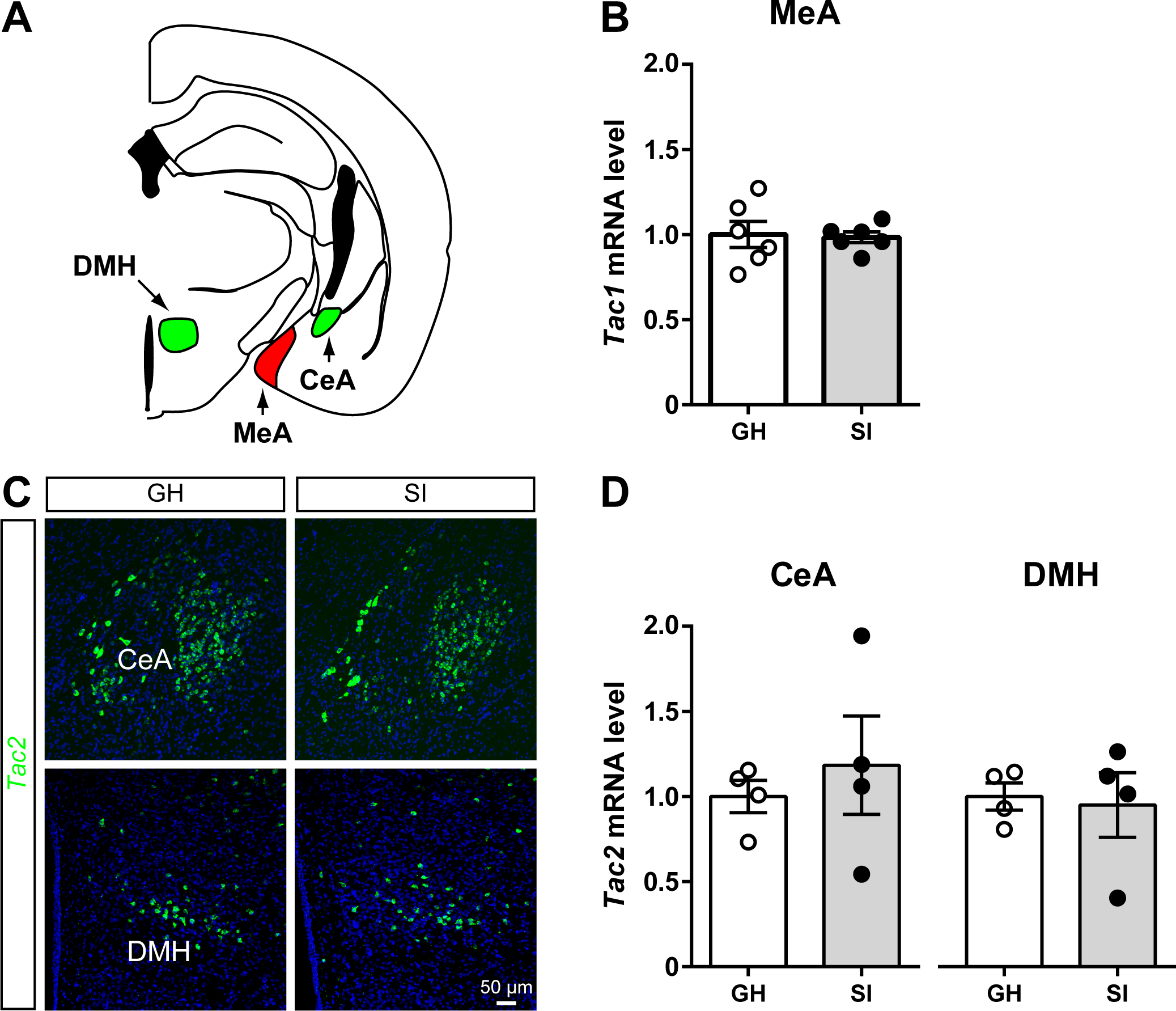


**Figure S4.** Prolonged social isolation does not alter the expression of *Tac1* and *Tac2* in the amygdala and hypothalamus.

**(A)** Schematic drawing depicting the dissected medial amygdala (MeA; red), central amygdala (CeA; green), and dorsomedial hypothalamus area (DMH; green). **(B)** Relative expression levels of *Tac1* mRNA in the MeA (n = 6) of socially isolated (SI) mice are presented as fold changes relative to that of group-housed (GH) mice. **(C)** Representative photographs of *in situ* hybridization for *Tac2* mRNA (green) in the CeA (upper) and DMH (lower) of GH and SI mice. Blue indicated DAPI staining of nuclei. **(D)** Relative expression levels of *Tac2* mRNA in the CeA (left; n = 4) and DMH (right; n = 4) of SI mice are presented as fold changes relative to that of GH mice. There is no significant difference in the *Tac1* and *Tac2* expression in the amygdala and hypothalamus between the two groups. Data are expressed as mean and SEM.


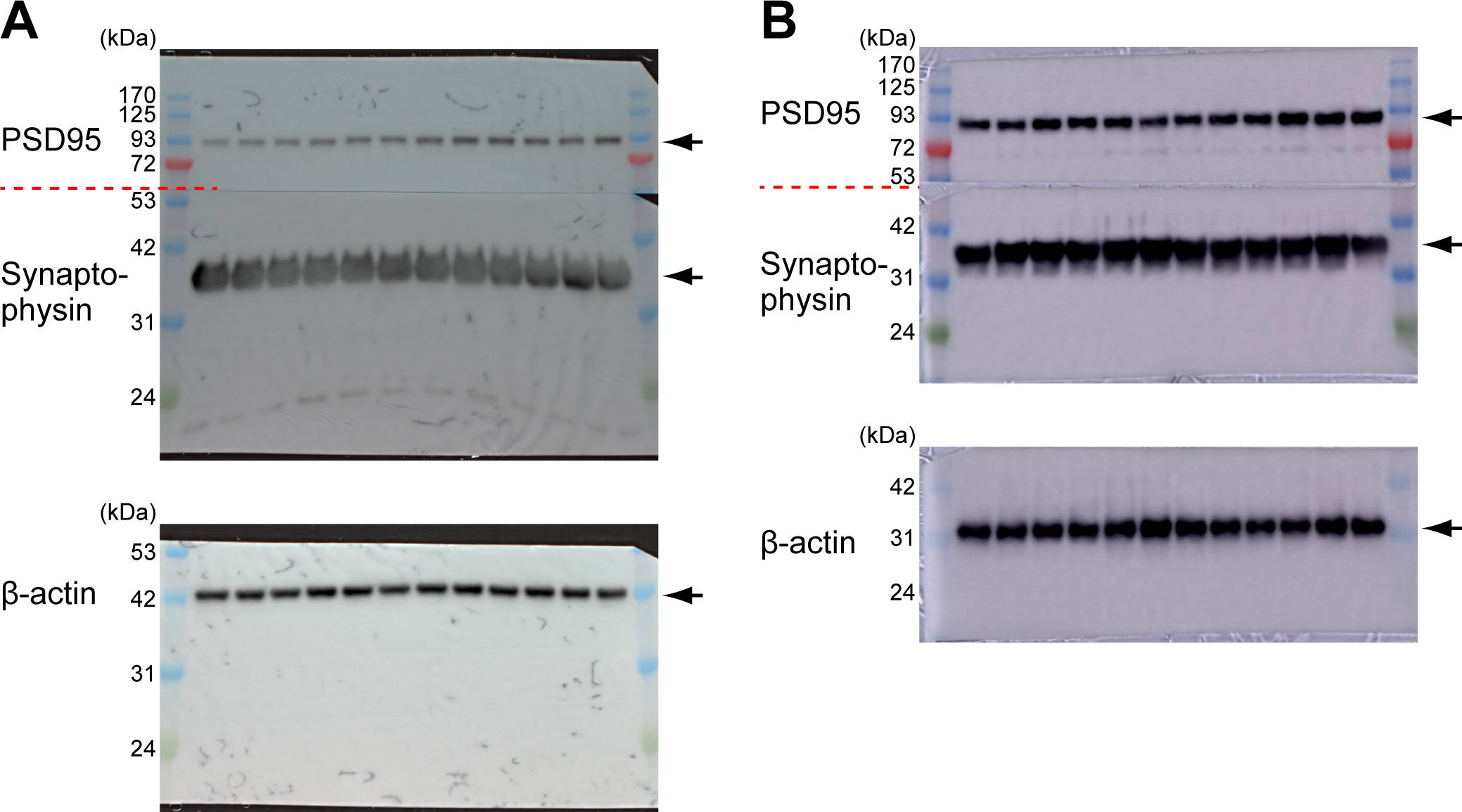


**Figure S5.** Raw data of western blots used in Figure 6A.

**(A, B)** Membranes cut horizontally above 53 kDa marker (red dashed line). The top portions of the membrane were probed for PSD95 and bottom portions were probed for synaptophysin and reprobed for β-actin.


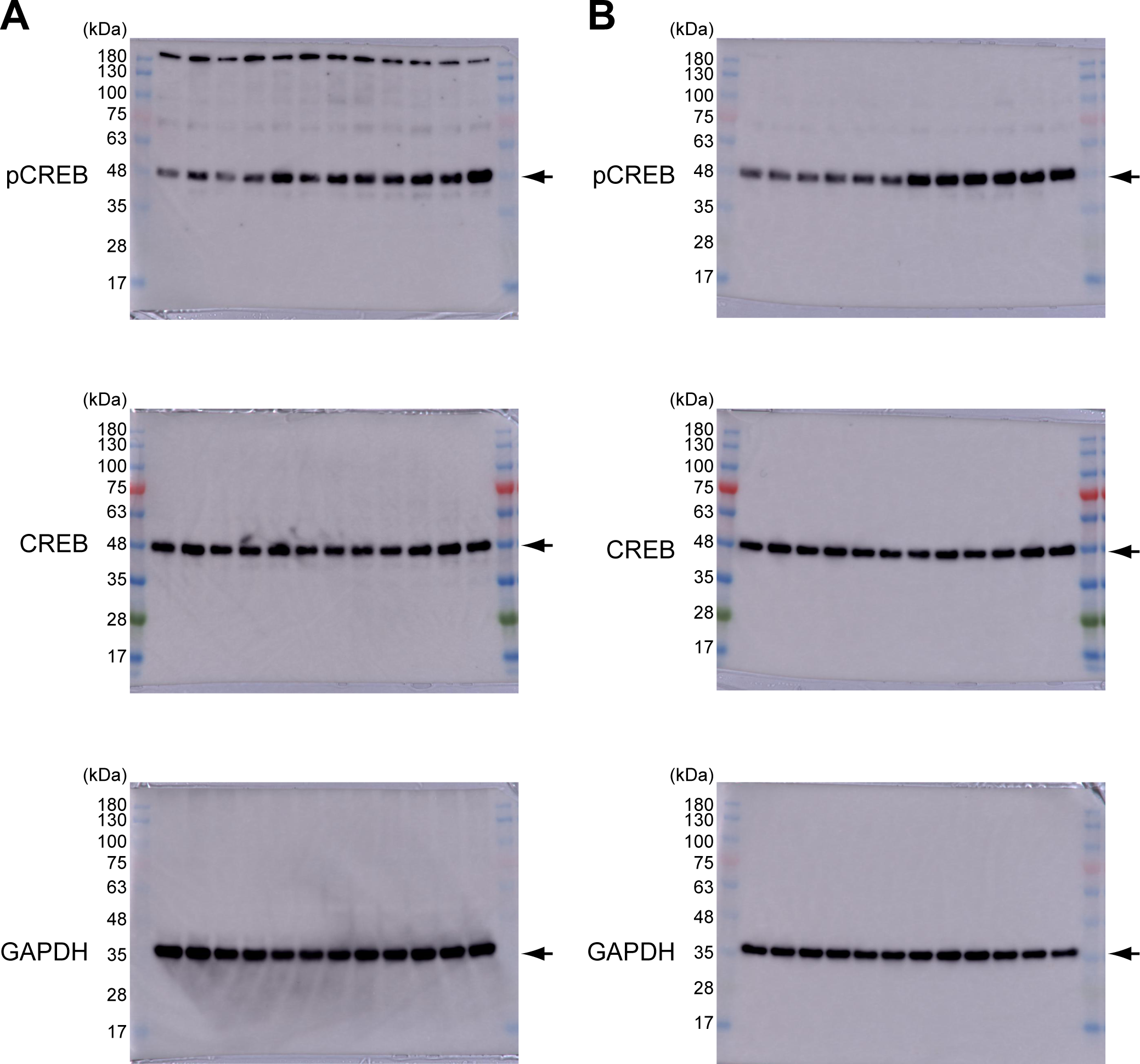


**Figure S6.** Raw data of western blots used in Figure 6B1.

**(A-C)** Membranes were probed for CREB (middle), stripped, and reporbed for phospho-CREB (pCREB; upper) and GAPDH (lower).

**
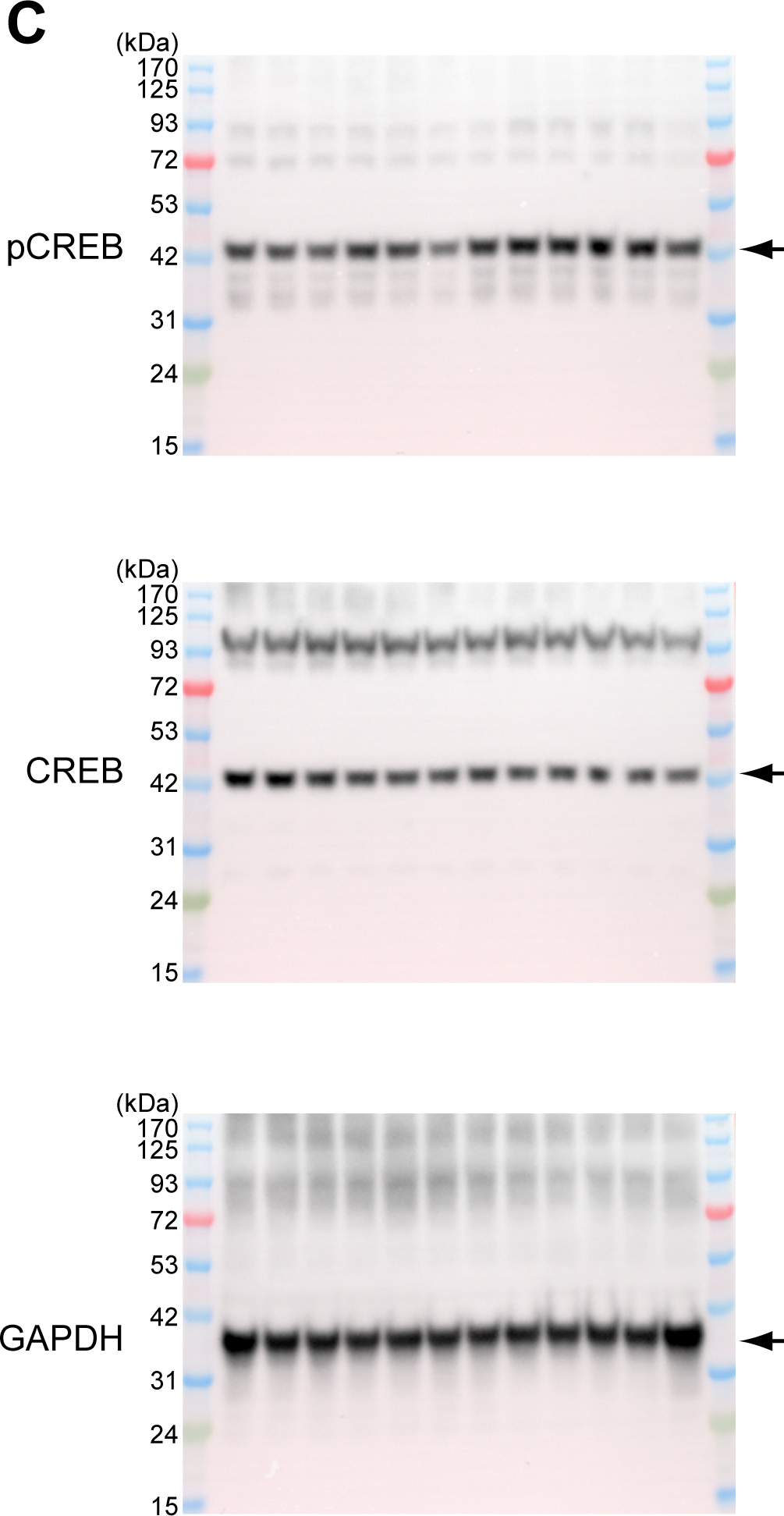
**

**Figure S6, continued.**


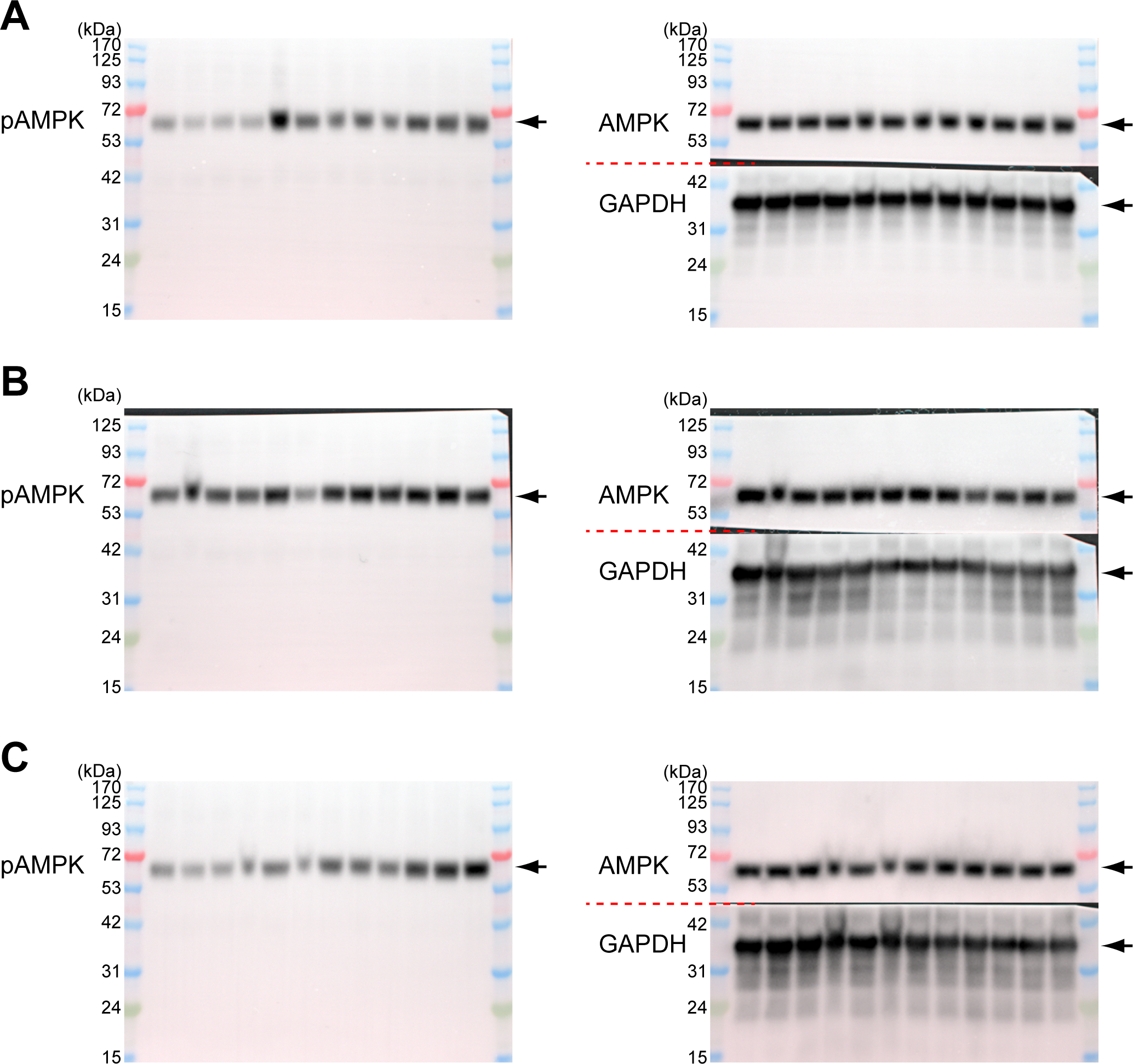


**Figure S7.** Raw data of western blots used in Figure 6B2.

**(A-C)** Membranes were probed for phosphor-AMPK (pAMPK; left), stripped, cut, and reporbed for AMPK (upper, right) and GAPDH (lower, right). Membrane cut horizontally below 53 kDa marker (red dashed line).
